# Intention to use artificial intelligence among SME account executives

**DOI:** 10.3389/frai.2026.1701133

**Published:** 2026-02-06

**Authors:** Gehad Mohammed Ahmed Naji, Muhammad Shoaib Saleem, Lim Cheah, Khairul Shafee Kalid, Yulita Hanum P. Iskandar

**Affiliations:** 1 Graduate School of Business, Universiti Sains Malaysia, USM, George Town, Malaysia; 2 INTI International University, Nilai, Malaysia; 3 Positive Computing Research Group, Universiti Teknologi PETRONAS, Seri Iskandar, Malaysia

**Keywords:** technology adoption and acceptance, artificial intelligence (AI), account executive, health system access, industrial growth, small and medium-sized enterprises (SMEs)

## Abstract

**Purposes:**

The study investigated the intention of account executives from Small and Medium Enterprises (SMEs) to employ artificial intelligence at their workplace. This study will examine the Unified Theory of Acceptance and Use of Technology (UTAUT), as well as technological and personal characteristics, and the role of SME account executives in adopting artificial intelligence. This study addresses the knowledge gaps in SME account executives’ understanding of artificial intelligence.

**Methodology:**

employed an online questionnaire distributed in collaboration with SMEs in Malaysia to gather responses from 273 account executives who work in SMEs. The data were analyzed using PLS-SEM and Artificial Neural Networks (ANN) to investigate SME account executives’ intentions to employ artificial intelligence. The demographic information of the individuals was analyzed using SPSS software.

**Results:**

The study’s findings revealed positive and significant relationships between performance expectancy, effort expectancy, social influence, facilitating conditions, system quality, employee awareness, and personal innovativeness toward artificial intelligence. Insignificant relationships were found between time-saving features and technological self-efficacy, and a negative, significant relationship existed with internet technology (IT) features toward artificial intelligence.

**Limitation:**

The cross-sectional approach focuses on SMEs in Malaysia, where the study’s applicability to other industries and countries is limited due to changes in the cultural, economic, and regulatory environment. Because participants may give socially acceptable answers rather than honest ones, using self-reported data raises the possibility of bias. Because inquiry assumes a certain level of knowledge with AI technology, respondents’ varying levels of digital competency may influence the findings.

**Practical implication:**

The findings of this study can help SMEs adopt artificial intelligence for their operations, particularly in accounting departments. Collaboration among organizations can help improve employee motivation to increase intention to use artificial intelligence.

**Originality/value:**

This study uses the Unified Theory of Acceptance and Use of Technology (UTAUT), technical qualities, and individual traits.

## 1 Introduction

Artificial intelligence (AI) is revolutionizing businesses by enhancing efficiency, fostering innovation, and transforming human–machine interactions (Chennupati and Kumar, 2024). While automation offers significant transformative potential, the human role remains critical in leveraging AI to achieve organizational success. While artificial intelligence is advancing swiftly, its implementation, especially among smaller businesses, remains an area with limited research. Research has not yet thoroughly examined the use of AI in SMEs (Borah et al., 2022; Zavodna et al., 2024; Ikpe, 2024; Mariani et al., 2023). A thorough analysis of the literature on AI reveals that a substantial percentage of published research focuses on AI applications in the healthcare and educational sectors (Bolanos et al., 2024; Jen and Salam, 2024; Kalid et al., 2024).

Malaysia’s national economy is primarily driven by 97% of small and medium-sized enterprises (SMEs) (Japar, 2024). However, many SMEs face persistent challenges that hinder their adoption of artificial intelligence (AI), including a scarcity of technical expertise, limited awareness, and insufficient resources (Jusoh and Ahmad, 2019). These issues are particularly evident in accounting departments, where only 30% of SMEs have implemented AI solutions, compared to other business functions that have embraced AI for operational improvements (Deinert et al., 2025; Faisal et al., 2023; Schwaeke et al., 2025).

**Figure 1:**
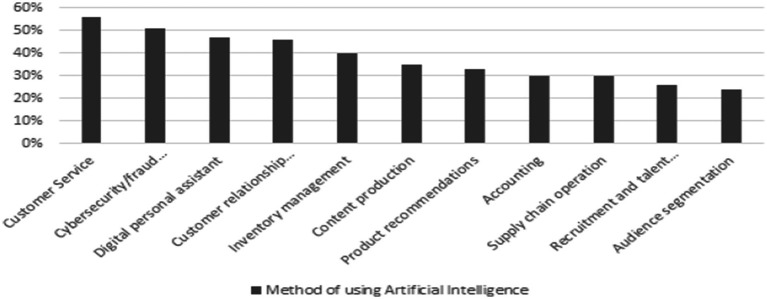
Global adoption of artificial intelligence among business owners: current use and future intentions (Lixandru, 2024; Riad, 2024).

**Figure 2:**
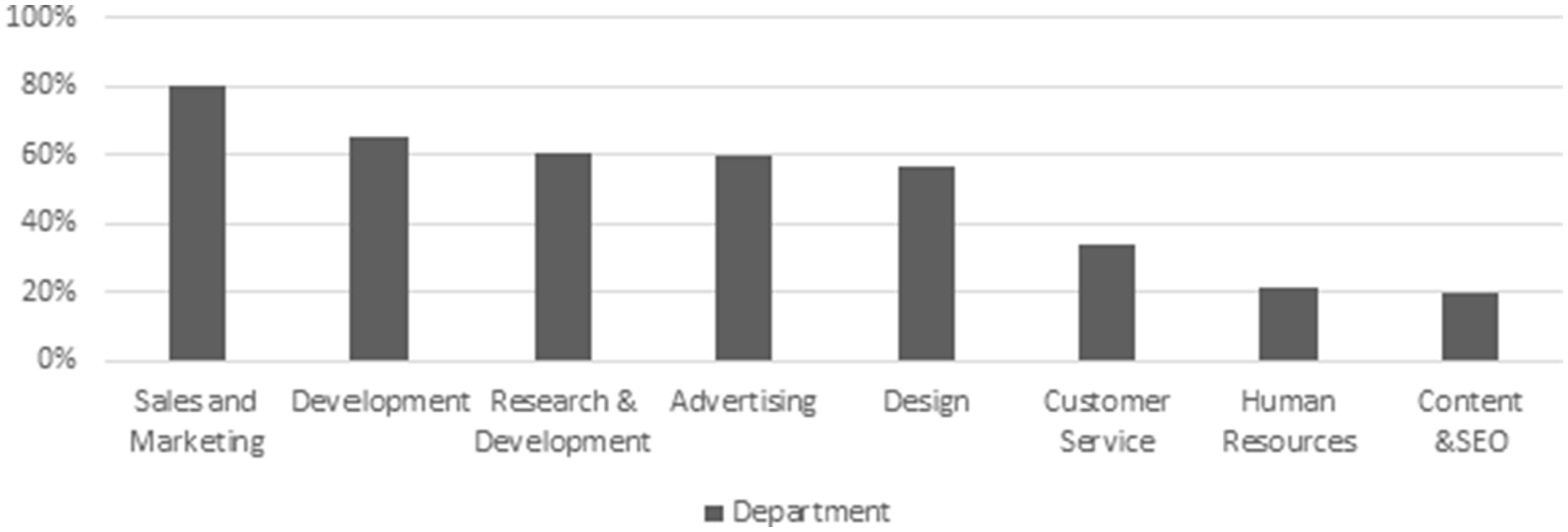
Key functional areas where SMES most commonly apply artificial intelligence (Wei and Pardo, 2022).

According to the Department of Statistics (2023), the account executive role ranks among the top three most sought-after job titles, underscoring its vital importance within organizations. Account executives play a crucial role in creating positive value for the company, particularly in SMEs (Popescu, 2020; Rukmana et al., 2022; Georges and Eggert, 2003). By overseeing financial operations, maintaining accuracy, and contributing to decision-making, account executives play a crucial role in adding value for SMEs (Popescu, 2020; Rukmana et al., 2022; Tauringana and Adjapong Afrifa, 2013). Nevertheless, a recent study found that nearly three-quarters (73%) of accounting professionals have not yet incorporated AI into their workflows (Zheng et al., 2024). This slow adaptation of AI into workflows demonstrates a significant gap in AI adoption within accounting roles, especially compared to other business departments that have embraced AI more readily, as shown in Figures 1, 2 (Lixandru, 2024; Riad, 2024; Wei and Pardo, 2022).

The Unified Theory of Acceptance and Use of Technology (UTAUT), a widely accepted model that explains user behavior and technology adoption through constructs such as performance expectancy, effort expectancy, social influence, and facilitating conditions, serves as the foundation for this study to overcome these obstacles (Venkatesh et al., 2003). However, additional constructs that consider the particular possibilities and barriers posed by AI must be incorporated to adapt the UTAUT model to the novel context of AI adoption among SME account executives (Venkatesh et al., 2003). Individual attributes (technological self-efficacy, employee awareness, and personal innovativeness) and technological attributes (IT features, system quality, and time-saving features) enhance the explanatory power of the UTAUT model. These attributes reflect user capabilities and system characteristics in shaping adoption behavior, particularly in resource-constrained environments like SMEs. For instance, technological self-efficacy determines users’ confidence in interacting with complex technologies, while IT features and system quality address usability, and reliability concerns crucial to AI adoption (Nirapai and Leelasantitham, 2024; Babar et al., 2025). Integrating these constructs aligns with prior recommendations to extend UTAUT by incorporating additional factors relevant to specific contexts (Venkatesh et al., 2012).

The purpose is to close the knowledge gap about how these characteristics influence SME account executives’ adoption of AI. Examining these factors provides valuable information for practitioners and policymakers to enhance accounting procedures, promote the use of AI in SMEs, and foster creativity and efficiency in Malaysian companies.

## 2 Literature review

A systematic literature review analysis was conducted on the application of technology and AI among SMEs, resulting in 315 articles that were filtered down to 13 publications, summarized in Appendices I, II. The outcome of this systematic review was instrumental in identifying key research gaps and informing the selection of constructs for the conceptual framework. Specifically, it highlighted the limited application of UTAUT in SME accounting contexts and the need to integrate additional technological and individual attributes to enhance explanatory power (Hallinger, 2013).

The UTAUT model shows strong explanatory power and effectively understands individuals’ willingness to use technology. It focuses on performance expectancy, effort expectancy, social influence, and facilitation conditions (Venkatesh et al., 2003; Jameel et al., 2023). It is a robust framework for studying individuals’ willingness to use technology (Venkatesh et al., 2003; Souiden et al., 2021).

However, UTAUT is limited when applied to innovations, especially AI in SMEs for account executives. The application of UTAUT for AI among account executives in SMEs has not been sufficiently explored (Le Dinh et al., 2025). UTAUT may not fully capture account executives’ intentions, as AI adoption in SMEs is often optional and context-specific (Souiden et al., 2021). There is a scarcity of academic contributions focusing on this specific context (Borah et al., 2022; Bouteraa et al., 2024; Oldemeyer et al., 2024; Pozzo et al., 2024; Polisetty et al., 2024). Additionally, it fails to consider the technological and individual factors that provide a strong basis for fully comprehending how customers behave toward AI (Jeyaraj et al., 2006).

Applying a new context to the UTAUT model will be critical in advancing theory (Venkatesh et al., 2012; Alvesson and Kärreman, 2007; Bouteraa et al., 2024; Hong et al., 2021). These factors are essential when exploring AI in smaller businesses, especially those that lack resources (Borah et al., 2022; Nascimento and Meirelles, 2022). Building on prior research Bouteraa et al. (2024), this study incorporates technological attributes (e.g., system quality, IT features, and time-saving features) and individual attributes (e.g., technological self-efficacy, employee awareness, and personal innovativeness) as additional constructs. These determinants are particularly relevant in the SME context, where resource constraints amplify the importance of technical reliability and individual readiness.

A strong theoretical framework for understanding people’s views about AI will be established by examining individual and technological characteristics. However, when incorporating AI into open innovation processes, it is essential to assess technical attributes such as system quality, IT features, time-saving features, and human oversight, including technological self-efficacy, employee awareness, and personal innovativeness (Jeyaraj et al., 2006; Bouteraa et al., 2021; Al-Daihani and Abdullah, 2023; Bin-Nashwan and Al-Daihani, 2021).

### 2.1 Theoretical background and hypothesis development

#### 2.1.1 Unified theory of acceptance and use of technology

The Unified Theory of Acceptance and Use of Technology (UTAUT) integrates eight prominent models of technology acceptance: the Theory of Reasoned Action (TRA), Social Cognitive Theory (SCT), Technology Acceptance Model (TAM), Theory of Planned Behavior (TPB), Motivational Model (MM), Model of PC Utilization (MPCU), Combined TAM and TPB (C-TAM-TPB), and Innovation Diffusion Theory (IDT) are the eight theoretical models that form the basis of UTAUT. It aims to determine whether a person voluntarily adopts and utilizes technology. The most thorough framework for explaining the dissemination of technology is the UTAUT model (Souiden et al., 2021). These constructs have been widely validated in various organizational and technological contexts, making UTAUT one of the most comprehensive frameworks for explaining the adoption of technology.

Although UTAUT2 includes additional constructs such as Hedonic Motivation, Price Value, and Habit, these were not incorporated in this study. This choice was made because UTAUT2 was primarily developed for consumer contexts. In contrast, this research focuses on an organizational setting—specifically, SME account executives—where cost-related decisions (e.g., Price-Value) are typically made at the managerial level. Moreover, constructs like Price Value are less relevant to individual users who are not directly responsible for financial investments in technology. Instead, this study extends the original UTAUT model by integrating context-specific technological and personal attributes that better reflect the operational realities and decision-making influences within SMEs.

Moderators such as age, gender, voluntariness of use, and experience have been excluded due to core contrasts that are more directly relevant to understanding the attitudes toward AI adoption in SMEs. Users will be positive when a technology meets their expectations (Venkatesh et al., 2003). Additionally, the original UTAUT model lacks integration of specific individual and technological factors that are critical in the context of AI adoption, particularly in resource-constrained environments such as SMEs (Jeyaraj et al., 2006).

##### 2.1.1.1 Performance expectancy

Performance expectancy in this study refers to the degree to which an account executive believes that implementing AI would improve their work efficiency. AI use in businesses has been associated with better performance and decision-making (Nascimento and Meirelles, 2022; Jameel et al., 2022). The ambition to employ technology increases with the number of advantages it may offer the workplace (Kwarteng et al., 2024). Performance expectations have a positive impact on the intention to utilize AI.

*H1*: Performance expectancy has a positive and significant impact on the intention to use AI among SME account executives.

##### 2.1.1.2 Effort expectancy

Effort expectation is related to how easily account executives may use AI in their workplace (Venkatesh et al., 2012). It is comparable to perceived ease of use, which reflects people’s perceptions of AI as uncomplicated and straightforward, requiring little mental or physical effort on their behalf (Venkatesh et al., 2003). The desire to utilize AI will be adversely affected by the usage of AI that is labor-intensive (Norzelan et al., 2024). When AI is readily incorporated into current work procedures, employees embrace technology (Nascimento and Meirelles, 2022). Because SMEs have limited time and resources, employees’ intentions will be influenced by how easily new technology is integrated and operated (Kwarteng et al., 2024). The aim to adopt the technology will be encouraged by its simplicity of use (Utomo et al., 2021). Consequently, the intention to embrace AI is positively influenced by the expectation of effort.

*H2*: Effort expectancy has a positive and significant impact on intention to use AI among SME account executives.

##### 2.1.1.3 Social influence

Social influence refers to the account executive’s perception that influential individuals believe AI should be deployed (Venkatesh et al., 2012). According to social influence theory, people’s behavior may be influenced by their group membership, which also increases the likelihood that they will adopt technologies and systems (Aziz et al., 2022). People constantly learn new technology from their peers to ensure they are included in the group (Terblanche and Kidd, 2022). Individuals in a collective society are continually influenced by the individuals in their social circles, including friends, family, and coworkers (Alam et al., 2020). Therefore, social influence has a positive effect on the intention to use AI.

*H3*: Social influence has a positive and significant impact on the intention to use AI among SME account executives.

##### 2.1.1.4 Facilitating condition

Facilitating conditions refer to the account executive’s perception that the organization’s infrastructure enables the practical application of AI. According to Kwarteng et al. Kwarteng et al. (2024), enabling settings can improve employees’ intention to use technology and reduce resistance. According to Sitthipon et al. Sitthipon et al. (2022), the best predictor of desire to utilize chatbots and applications is conducive conditions (Naji et al., 2025a). The market needs to consider the enabling circumstances. When the current technological infrastructure is easy to use and promotes the use of AI, employees are more likely to accept it (Hallinger, 2013). Technical support that encourages the intention to use new technology and assists with troubleshooting also plays a role (Menon and Shilpa, 2023). Companies should provide the necessary conditions, resources, and infrastructure to facilitate the smooth adoption of AI (Wongras and Tanantong, 2023).

*H4*: Facilitating condition has a positive and significant impact on the intention to use AI among SME account executives.

#### 2.1.2 Technological attributes

The technology of accounting offers features such as user interface, functionality, security, and time-saving capabilities, which are critical determinants in the adoption process. When people believe technology is simple, safe, and time-efficient, they are more likely to use it (Al-Daihani and Abdullah, 2023; Bouteraa et al., 2023).

##### 2.1.2.1 Internet technology feature

With the advancement of technology, SMEs have incorporated internet technology (IT) into their operations. Internet technology (IT) features, including its security measures, privacy safeguards, reliability, and effectiveness, will determine the user’s choice of technology (Sura et al., 2017). The widespread adoption of technology is likely to be influenced by information technology, as online capabilities enhance efficiency and boost workplace productivity. Users can save time and improve performance by leveraging digital solutions for various tasks (Al-Daihani and Abdullah, 2023; Bin-Nashwan and Al-Daihani, 2021; Bouteraa et al., 2023). AI’s power can help account executives increase efficiency and productivity, as well as enhance data analysis and prediction (Johnson, 2023; Ghaleb et al., 2023). These features will help boost the acceptance of AI, as most account executives tend to be late adopters compared to other departments. Failure to understand people’s beliefs about internet features will negatively impact their attitudes toward technology (Sura et al., 2017).

*H5*: Internet Technology (IT) features have a positive and significant impact on the intention to use AI among SME account executives.

##### 2.1.2.2 System quality

System quality is seen as a crucial strategic element in the IS success model (DeLone and McLean, 1992). According to DeLone and McLean (2003), technology users expect features such as usability, availability, reliability, flexibility, and accessibility. The system’s quality significantly impacts people’s intentions to utilize technology (Bouteraa et al., 2024; Bouteraa et al., 2023; Bouteraa and Al-Aidaros, 2020; Ackerman et al., 2022; Anggreni et al., 2020). Better system quality will lead to increased system usage. High-quality features increase the likelihood that users will trust and plan to utilize a system. However, system quality varies depending on the account executive’s needs, preferences, and organizational requirements. According to user preferences, AI is a safe and effective tool for account executives to utilize for a variety of activities. In view of the above, the following hypothesis is formed for assessment:

*H6*: System quality positively and significantly impacts the intention to use AI among SME account executives.

##### 2.1.2.3 Time saving feature

Implementing the system can enhance efficiency, decreasing the duration required for task completion (Dazmin and Ho, 2019). Time is essential for everyone. In our fast-paced contemporary society, time is a crucial intangible asset that we must exchange for other resources, such as wealth and effort (Xu et al., 2019). Employees have consistently been motivated to embrace technology due to the increased productivity resulting from time-saving benefits (Bodea et al., 2024; Yapp and Kataraian, 2022). Incorporating AI technology into workplace processes can enhance efficiency and goal attainment by providing timely and accurate information. For account executives, AI can conserve time by automating routine tasks, improving customer service efficiency, and facilitating training (Bodea et al., 2024). In view of the above, the following hypothesis is formed for assessment:

*H7*: The time-saving feature positively and significantly impacts the intention to use AI among SME account executives.

#### 2.1.3 Individual attributes

People’s attitudes significantly influence the desire to adopt AI in SMEs. Their perceptions of AI’s benefits and how well it aligns with their values influence their readiness to embrace it. Because AI aligns with their ideas and fundamental values, people who respect sustainable practices will be more inclined to use it (Bouteraa et al., 2023).

##### 2.1.3.1 Technology self-efficacy

The social cognitive theory is the source of this idea (Bandura, 1989). In the context of technology, it refers to a person’s technical self-efficacy, or confidence in their ability to use technology to complete a task. In the face of difficulties, confidence is the most critical factor in facing challenges in many positions (Ariffien et al., 2023). AI technology has the potential to reduce employees’ daily workload, provide performance-related insights, and boost their self-confidence in using the system (Rao et al., 2024; Leggatt, 2023; Liwanag and Galicia, 2023). In view of the above, the following hypothesis is formed for assessment:

*H8*: Technology self-efficacy positively and significantly impacts the intention to use AI among SME account executives.

##### 2.1.3.2 Employee awareness

Employee awareness pertains to their understanding of a specific subject matter. Insufficient technological awareness can diminish the propensity to employ a system (Bouteraa et al., 2021). A well-informed public will be more conscious of and inclined to support a given service (Lujja et al., 2018). Numerous researchers have underscored the importance of awareness in shaping intention (Flavián et al., 2020; Zirar et al., 2023). The level of technological awareness among employees will affect their intention to use it. In view of the above, the following hypothesis is formed for assessment:

*H9*: Employee awareness has a positive and significant impact on the intention to use AI among SME account executives.

##### 2.1.3.3 Personal innovativeness

The capacity of people or organizations to persuade others to utilize the technology. Individuals who possess a high degree of personal inventiveness will have a strong viewpoint on cutting-edge technology. This personal innovative trait plays a vital part in adopting new ideas and technologies (Abbasi et al., 2021). Individuals with high levels of innovation are more likely to adopt new concepts or innovations (Kandoth and Shekhar, 2022). The extent of personal innovativeness is gaged by an individual’s eagerness to engage in novel activities (Ciftci et al., 2021). A person with high innovativeness will be an early adopter of technologies and influence others to adopt them as well (Bouteraa et al., 2024). Those more receptive to innovation are more inclined to utilize new systems. The possible reason is that they will see the value of the new technology and be willing to change their habits (Kandoth and Shekhar, 2022). Aforementioned in view, the following hypothesis is formed for validation:

*H10*: Personal Innovativeness positively and significantly impacts the intention to use AI among SME account executives.

### 2.2 Theoretical framework

**Figure 3:**
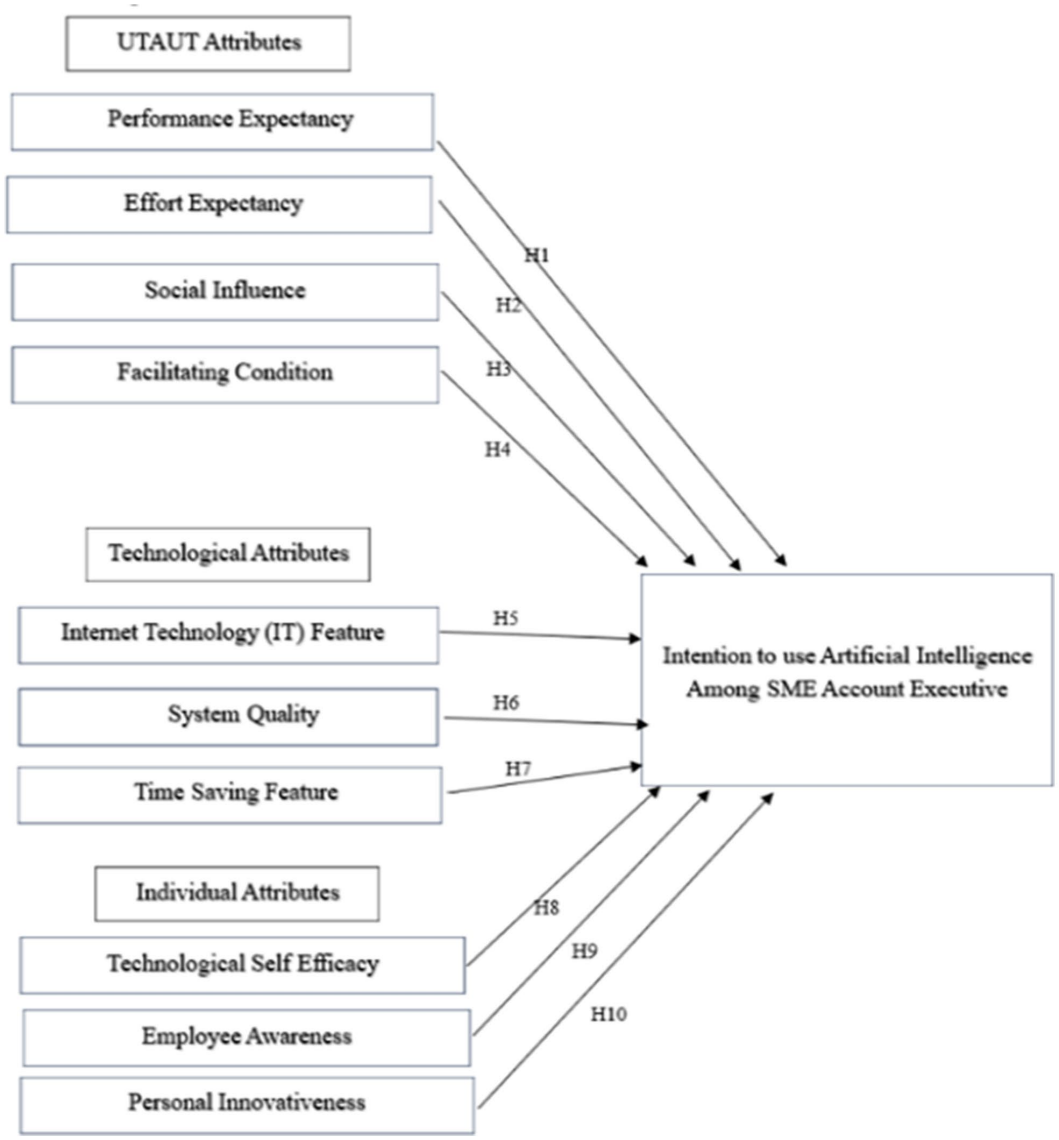
Proposed conceptual framework.

To investigate the intention to utilize AI among SME account executives, this study employs an extended version of the UTAUT, incorporating technological characteristics and individual qualities (Figure 3).

## 3 Research methodology

### 3.1 Research design

The survey design is straightforward and will serve as an excellent research tool for the paper. Survey design is a method that can answer the research question, which includes describing, comparing, and identifying the relationship between the variables (Creswell and Creswell, 2017). This research will use a quantitative cross-sectional survey to collect the data from account executives among SMEs. The study aims to understand SME account executives’ intentions to adopt AI technologies, utilizing the UTAUT framework. A snapshot of current attitudes and perceptions was sufficient to explore these objectives and address the research questions. Data will be collected only once using a structured questionnaire to address this research question. Therefore, a cross-sectional design cannot determine whether one variable causes another (Taris et al., 2021).

### 3.2 Survey instrument

The instruments of structure or format are classified as questionnaires and scales (Sathiyaseelan, 2015). Using a five-point Likert scale, drawing inspiration from previous research. The main content of the questionnaire will be preceded by a cover letter outlining the purpose and study title. It also includes four screening questions before proceeding to Section A. This questionnaire consists of two sections, each with 55 items, labeled as Sections A and B, and is attached in Appendix IV: Questionnaire. Section A aims to collect demographic information, including gender, age, state, and monthly income. Section B for the research instrument will focus on the variables relevant to AI among SME account executives and use a Likert scale of 1 to 5 for the responses, which are Strongly Disagree (1), Disagree (2), Neutral (3), Agree (4), and Strongly Agree (5). It is adapted from prior studies, including (Venkatesh et al., 2003; Bouteraa et al., 2023; Sura et al., 2017; Akbar et al., 2023; Saville and Foster, 2021). The measurement items for each construct were adapted from validated scales in earlier studies. Specifically, Performance Expectancy, Effort Expectancy, Social Influence, and Facilitating Conditions were based on Venkatesh et al. (2003); Internet Technology Features from Bouteraa et al. (2023); System Quality from DeLone and McLean (1992); Time-Saving Feature from Dazmin and Ho (2019); Technological Self-Efficacy from Bandura (1989), Bodea et al. (2024), and Yapp and Kataraian (2022); Employee Awareness from Lujja et al. (2018), Rao et al. (2024), Leggatt (2023), and Liwanag and Galicia (2023), and Personal Innovativeness from Agarwal and Prasad (1998). To enhance transparency and facilitate the assessment of construct validity and potential overlap between conceptually similar variables (e.g., Time-Saving Feature and Effort Expectancy), the complete questionnaire used for data collection is included in Appendix IV.

### 3.3 Sampling procedure

Individual account executives among Malaysian SMEs are the subject. Purposive sampling, a non-probability technique, is employed in the study to select participants based on predetermined criteria. According to Tongco (2007) notes that purposive sampling involves strategically selecting participants to ensure they meet the study’s objectives, rather than using random selection. Although probability sampling is considered ideal, it may be impractical due to time, resource, and cost constraints (Achim et al., 2024).

The sample size was determined through a power analysis for a multiple regression model using G*Power software, version 3.1.9.7. According to the study, a minimum sample size of 118 was required, based on a medium effect size of 0.15, a 0.05 significance level (*α*), 0.80 statistical power (1-*β*), and 10 predictors (Grüner, 2020). This sample size ensures adequate statistical power to detect meaningful associations in the data. This study achieved an actual response rate of 273, surpassing the necessary level to improve reliability and consider any non-response bias.

Participants are selected based on the following criteria: Participants were screened using four preliminary questions to ensure they met the inclusion criteria related to employment status, experience, prior AI usage, and basic AI knowledge.

1. Account executives are currently employed by SMEs located in Malaysia.
2. At least 1 year of professional experience in SMEs.
3. No prior use of AI in their professional roles.
4. Demonstrated knowledge of AI technology.

A brief pre-survey assessment was administered to measure participants’ knowledge of AI, verifying their familiarity with basic AI concepts and terminology. This pre-survey ensured that only participants with relevant background knowledge were included in the study.

### 3.4 Statistical analysis

SPSS was used for demographic and ANN analysis. The measurement, structural model, and analysis were conducted using SMARTPLS SEM. The study consisted of two primary stages: evaluating measurement models and structural models. These metrics were chosen based on previous research and applied in accordance with the recommendations (Hair et al., 2021). SPSS was utilized for demographic analysis to identify the significance and construct in the research model (Elareshi et al., 2022).

The ANN is also an appropriate tool for measuring operations in complex and nonlinear input–output scenarios (Liébana-Cabanillas et al., 2018). An ANN model was constructed using the feature scores from the PLS-SEM model to validate the proposed relationships in the research model. The ANN architecture consists of a multi-layer perceptron utilizing feed-forward and backpropagation (FFBP) techniques. Error and input both spread backwards and forward, accordingly. The deep ANN structure consists of two hidden layers with many neurons between the input and output layers (Mishra et al., 2024). In addition, the application of ANN, which has lately become widely used in information systems, is one of the models studied in the AI approach (Almarzouqi et al., 2022a). It has also been applied in technology acceptance and adoption studies (Elareshi et al., 2022; Almarzouqi et al., 2022b; Akour et al., 2022). This application is notable for its ability to outperform other modeling tools, such as linear modeling, regression, and PLS-SEM.

## 4 Results

### 4.1 Multivariate data distribution

Both univariate and multivariate methods were used in the data distribution study to evaluate skewness and kurtosis. Normality plays a significant role in both univariate and multivariate models. The Web Power online calculator was used to calculate Mardia’s multivariate skewness and kurtosis (Sutrisno, Nirwana, and Wulandari, 2021) (Wulandari et al., 2021). Univariate skewness and kurtosis were calculated using SPSS software. According to Appendix VI, the univariate skewness and kurtosis were computed.

The acceptable range for univariate normality is ≤ ± 2 for univariate skewness and ≤ ± 7 for univariate kurtosis (Curran et al., 1996). The acceptable range for multivariate normality is ≤ ± 3 for multivariate skewness and ≤ ± 20 for multivariate kurtosis (Kline, 2023). Table 1 showed kurtosis (184.34), whereas skewness was −17.54. Therefore, the researcher concluded that univariate normality is acceptable, whereas multivariate normality was not achieved. Consequently, this research used PLS-SEM (Shmueli et al., 2019). While skewness and kurtosis were examined during the data screening process to assess the distribution of the dataset, it is acknowledged that PLS-SEM does not require multivariate normality for model estimation or for evaluating validity metrics such as AVE, CR, *R*^2^, or path coefficients. Therefore, the normality assessment was conducted solely for descriptive purposes and not as a prerequisite for the PLS-SEM analysis.

**Table 1:** Mardia’s multivariate skewness and kurtosis.

Statistic	*B*	*z*	*p*-value
Skewness	17.74832	807.54874	0
Kurtoses	183.42822	19.74935	0

### 4.2 Common method variance test

The same source of data, namely online survey questionnaires, was used in this study. The correlation matrix between the components in the study model was examined using a complete collinearity test (Kock and Lynn, 2012). VIF levels below five may be deemed appropriate (Kock, 2015). Every VIF value in Table 2 was less than 5.

**Table 2:** Full collinearity statistics (VIF).

Construct	PE	EE	SI	FC	IT	SQ	TSF	TSE	EA	PI
VIF	3.244	3.793	2.300	3.418	2.030	3.321	3.036	3.264	3.078	3.202

PE, Performance Expectancy; EE, Effort Expectancy; SI, Social Influence; FC, Facilitating Condition; IT, Internet Technology Feature; SQ, System Quality; TSF, Time Saving Feature; TSE, Technological Self-Efficacy; EA, Employee Awareness; PI = Personal Innovativeness.

### 4.3 Respondent profile

Account executives employed by SMEs in Malaysia were given access to the research questionnaire through an online survey. This study was conducted across the states of Malaysia, with a total of 500 surveys distributed and 424 responses received from August 11, 2024, to October 13, 2024. Additionally, 82 responses were deemed invalid as the respondents failed to answer the filtering question correctly. The final dataset consisted of 273 valid replies after 69 extra responses were eliminated using the standard deviation (SD) approach for data cleaning to ensure construct validity in the model (Bhuiyan et al., 2025). As a result, 54.60% of respondents completed this survey, above the 40% cutoff (Sataloff and Vontela, 2021). A high response rate increases the reliability and validity of data results (Wu et al., 2022). The survey from 273 respondents is presented in Table 3, with the majority being female (65.20%), while males constitute 34.80%. The largest age group was 25–34 years old (43.20%), followed by 18–24 years old (32.60%) and 35–44 years old (16.80%). The monthly income reported was with the majority in the RM2001-RM5000 (54.20%), above RM5000 (41.00%), and below RM2000 (4.80%). Geographically, the majority of respondents were from Selangor (27.80%), Johor (19.0%), and Pulau Pinang (12.10%).

**Table 3:** Demographic profile of respondents.

Demographic features	Frequency	Per cent	Cumulative percent
Respondent gender			
Female	178	65.20	65.20
Male	95	35.80	100
Respondent age group			
18–24	89	32.60	32.60
25–34	118	43.20	75.80
35–44	46	16.80	92.70
45–54	12	4.40	97.10
55 and above	8	2.90	100.00
Respondent monthly income			
Below RM2000	13	4.80	4.80
RM2001–RM5000	148	54.20	59.00
Above RM5000	112	41.00	100.00
Respondent state			
Johor	52	19.0	19.00
Kedah	27	9.90	28.90
Kelantan	4	1.50	30.40
Melaka	9	3.30	33.70
Negeri Sembilan	11	4.00	37.70
Pahang	8	2.90	40.70
Perak	16	5.90	46.50
Perlis	3	1.10	47.60
Pulau Pinang	33	12.10	59.70
Sabah	10	3.70	63.40
Sarawak	16	5.90	69.20
Selangor	76	27.80	97.10
Terengganu	8	2.90	100.00

### 4.4 Assessment of measurement model

All constructs in the proposed model were conceptualized and measured as reflective constructs. Accordingly, the assessment of the measurement model focused on evaluating internal consistency reliability (using Cronbach’s Alpha and Composite Reliability), convergent validity (using the average variance extracted, AVE), and discriminant validity (using the Heterotrait-Monotrait Ratio, HTMT), in line with guidelines for reflective measurement models in PLS-SEM (Achim et al., 2024). The measuring model was evaluated using convergent Validity, discriminant Validity, internal consistency, and reliability. While reliability was assessed using Composite Reliability (CR), Validity was analyzed using convergent and discriminant validity methods. Each indicator’s Composite Reliability is required to be more than 0.70 (Hair et al., 2021). Such findings indicate that every build satisfied the requirements for consistent dependability. All the outer loadings must be more than 0.70 for convergent Validity to hold. All constructs had to have average variance extracted (AVE) values more than 0.50 (Hair et al., 2021). All constructions met the criteria for consistent dependability, as shown in Table 4, which indicated that all CR values exceeded the 0.70 cutoff. The average variance extracted (AVE) for every construct was more than 0.50. According to research, each construct accounted for over half of the variation in the indicators (Hair et al., 2021). The convergent Validity of each of these indicators was verified.

**Table 4:** Construct reliability and validity.

Constructs	Items	Loadings	Cronbach’s alpha (CA)	Composite reliability (CR)	Average variance extracted (AVE)
Performance expectancy (PE)	PE1	0.922	0.932	0.949	0.787
	PE2	0.875			
	PE3	0.892			
	PE4	0.873			
	PE5	0.872			
Effort expectancy (EE)	EE1	0.887	0.912	0.938	0.790
	EE2	0.904			
	EE3	0.877			
	EE4	0.889			

Constructs	Items		Loadings	Composite reliability (CR)	Average variance extracted (AVE)
Social influence (SI)	SI1	0.832	0.895	0.917	0.613
	SI2	0.729			
	SI3	0.761			
	SI4	0.754			
	SI5	0.765			
	SI6	0.817			
	SI7	0.815			
Facilitating condition (FC)	FC1	0.886	0.901	0.931	0.771
	FC2	0.896			
	FC3	0.848			
	FC4	0.881			
Internet technology feature (IT)	IT1	0.89	0.911	0.933	0.737
	IT2	0.875			
	IT3	0.832			
	IT4	0.854			
	IT5	0.84			
System quality (SQ)	SQ1	0.882	0.930	0.947	0.782
	SQ2	0.867			
	SQ3	0.902			
	SQ4	0.873			
	SQ5	0.897			

Constructs	Items	Loadings	Composite reliability (CR)	Average variance extracted (AVE)	Constructs
Time saving feature (TSF)	TSF1	0.897	0.890	0.924	0.752
	TSF2	0.866			
	TSF3	0.856			
	TSF4	0.849			
Technology self-efficacy (TSE)	TSE1	0.858	0.891	0.92	0.697
	TSE2	0.824			
	TSE3	0.826			
	TSE4	0.815			
	TSE5	0.851			
Employee awareness (EA)	EA1	0.847	0.895	0.923	0.706
	EA2	0.853			
	EA3	0.872			
	EA4	0.844			
	EA5	0.782			
Personal innovativeness (PI)	PI1	0.855	0.851	0.900	0.692
	PI2	0.797			
	PI3	0.848			
	PI4	0.826			
Intention to use (IU)	IU1	0.911	0.866	0.918	0.788
	IU2	0.867			
	IU3	0.885			

One technique used to evaluate the relationship between latent variables is discriminant Validity. For discriminant Validity, the heterotrait-monotrait ratio (HTMT) was employed, as suggested by Hair et al. (2021) and Henseler et al. (2015). The cutoff value is below 0.90. A 0.90 or above would indicate the absence of discriminant Validity (Hair et al., 2021; Henseler et al., 2015). For data purification, it was therefore essential to use the standard deviation (SD) with a value less than 0.25, which resulted in the removal of 69 disengaged data sets (Bhuiyan et al., 2025). Table 5 demonstrates that every HTMT result was less than 0.90. In Table 5, it is shown that all HTMT values were below 0.90. Table 5 revealed that all HTMT values fell below 0.90, indicating participants perceived the 11 constructs used in the study as distinct. Thus, this research concludes that the reliability and Validity of this research were statistically validated.

**Table 5:** Discriminant validity (HTMT criterion).

Constructs	PE	EE	SI	FC	IT	SQ	TSF	TSE	EA	PI	IU
PE											
EE	0.805										
SI	0.703	0.712									
FC	0.784	0.852	0.743								
IT	0.658	0.712	0.629	0.650							
SQ	0.797	0.802	0.710	0.786	0.632						
TSF	0.767	0.799	0.712	0.789	0.656	0.774					
TSE	0.757	0.811	0.671	0.815	0.609	0.797	0.820				
EA	0.782	0.780	0.710	0.756	0.654	0.772	0.792	0.810			
PI	0.796	0.832	0.723	0.803	0.723	0.807	0.815	0.814	0.832		

### 4.5 Path coefficient and hypotheses summary

The path coefficient, standard error values, and *p*-values for the structural model were reported using a bootstrapping resampling approach with a sample size of 10,000 (Ramayah et al., 2018). Table 6, which summarizes the significance and correlation between the variables, displays the path coefficients. For H1, H2, H3, H4, H6, H9, and H10, the T values for one-tailed tests at a 95% significance level revealed p-values less than 0.05%. Thus, support was given to H1, H2, H3, H4, H6, H9, and H10. Hypotheses H5, H7, and H8 were rejected, though.

**Table 6:** Path coefficient and confidence interval.

Hypothesis	Relationship	Std. Beta	Std. error	*t*-values	*p* values	BCI LL	BCI UL	*F* ^2^	Magnitude	VIF	Result
HI	PE → IU	0.19	0.055	3.493	0	0.101	0.281	0.057	Small	3.244	Yes
H2	EE → IU	0.121	0.062	1.952	0.025	0.019	0.221	0.020	Small	3.793	Yes
H3	SI → IU	0.119	0.048	2.485	0.006	0.04	0.197	0.031	Small	2.300	Yes
H4	FC → IU	0.158	0.050	3.127	0.001	0.075	0.239	0.037	Small	3.418	Yes
H5	IT → IU	−0.071	0.040	1.78	0.038	−0.137	−0.005	0.013	No effect	2.030	No
H6	SQ → IU	0.117	0.057	2.043	0.021	0.023	0.210	0.021	Small	3.321	Yes
H7	TSF → IU	0.070	0.051	1.361	0.087	−0.015	0.154	0.008	Small	3.036	No
H8	TSE → IU	0.063	0.050	1.255	0.105	−0.021	0.143	0.006	Small	3.264	No
H9	EA → IU	0.124	0.051	2.422	0.008	0.04	0.207	0.025	Small	3.078	Yes
H10	PI → IU	0.137	0.052	2.612	0.005	0.049	0.221	0.030	Small	3.202	Yes

### 4.6 Assessment of structural model

The coefficient of determination is also known as the R-squared value. It calculates the percentage of the dependent variable’s variation that can be accounted for by the model’s independent variable (Achim et al., 2024). *R*^2^ was used to measure the model’s fit quality in the PLS-SEM framework (Cohen, 2013). According to Hair et al. (2021), the interpretation of *R*^2^ values varied, with 0.75, 0.50, and 0.25 often considered considerable, moderate, and weak, respectively. However, the research environment determined the acceptable *R*^2^ values (Hair et al., 2011). *R*^2^ value for intention to use was substantial. The more predictor constructs were included, the higher the *R*^2^ value became (Hair et al., 2021). The result shown in Table 7 is considerable. *R*^2^ values of 0.804 represented that 80.40% of the variance of the dependent variable (Intention to use) was explained by the independent variable (performance expectancy, effort expectancy, social influence, facilitating condition, internet technology feature, system quality, time-saving feature, technological self-efficacy, employee awareness, and personal innovativeness) in the model. A higher *R*^2^ value suggested a better fit of the model to the data (Hair et al., 2011).

**Table 7:** *R*^2^ Coefficient of determination.

Construct	*R*-square
Intention to use	0.804

The *R*^2^ statistics were interpreted by several studies as an indicator of the predictive strength of the model (Shmueli and Koppius, 2011). *R*^2^ just showed the model’s explanatory power within the sample; it said nothing about the model’s predictive capacity, hence this interpretation was not entirely correct (Hair et al., 2011; Chin et al., 2020). PLS predict was created as an out-of-sample prediction method to overcome this constraint (Shmueli et al., 2019). This method uses a 10-fold process and the PLS-Predict measurement strategy. A model’s predictive significance is shown by a *Q*^2^ value larger than “0” (Shmueli et al., 2019). The more appropriate metric for prediction was thought to be RMSE (Shmueli et al., 2019). A comparison between the RMSE and the naïve LM benchmark is shown in Table 8. Researchers used RMSE in accordance with the guidelines (Shmueli et al., 2019). The PLS model’s (PLS-RMSE) errors were all less than the LM model’s (LM-RMSE) errors. Q > 0 in Table 8 and a high degree of predictive power were attributes of the model employed in this investigation.

**Table 8:** PLS predict.

Construct	*Q*^2^ predict	PLS-SEM_RMSE	PLS-SEM_MAE	LM_RMSE	LM_MAE	IA_RMSE	IA_MAE
IU1	0.649	0.798	0.635	0.813	0.632	1.346	1.129
IU2	0.576	0.711	0.574	0.758	0.614	1.092	0.913
IU3	0.622	0.707	0.577	0.760	0.617	1.15	0.947

IU, Intention to use.

### 4.7 Effect size (*f*^2^)

**Figure 4:**
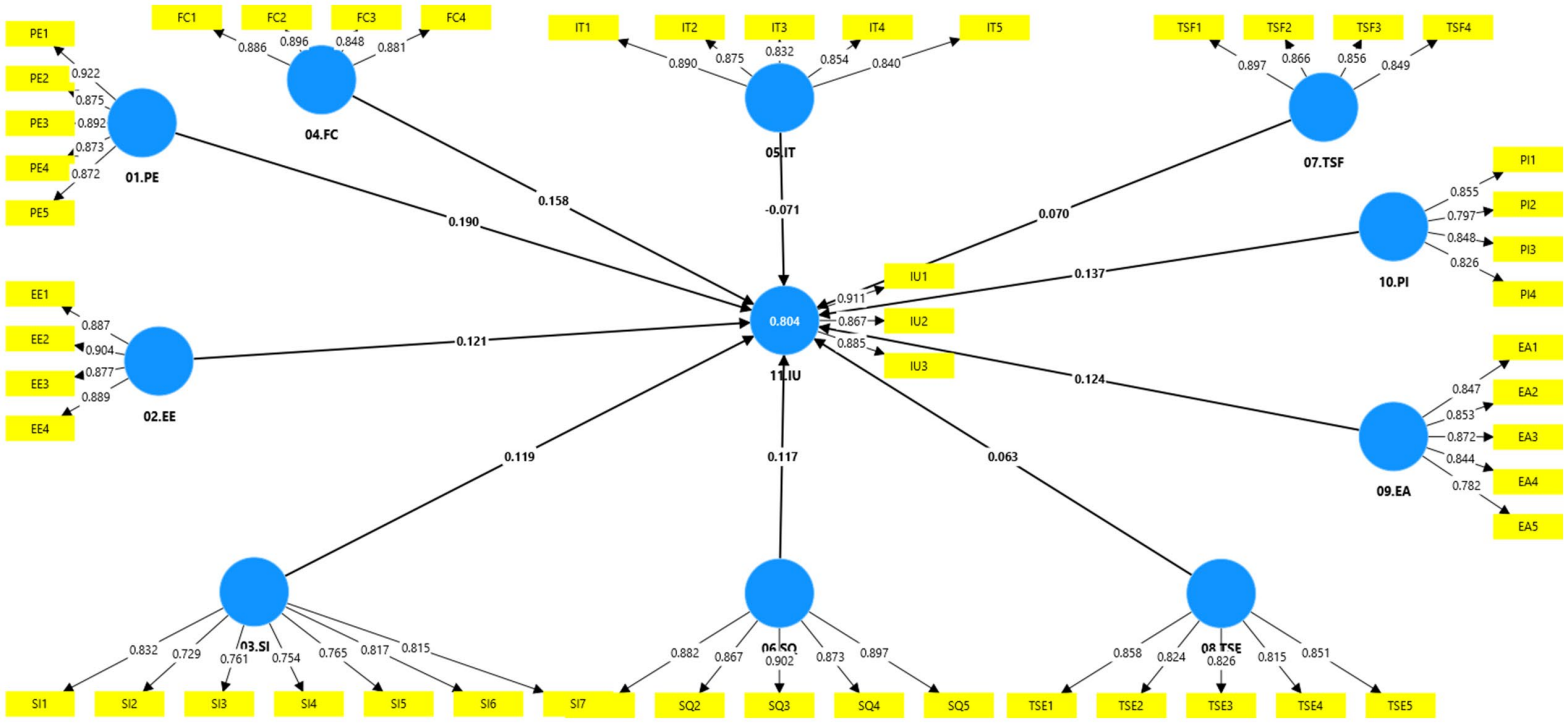
PLS structure model.

The route coefficients and f^2^ are comparable. Although it wasn’t necessary, it may offer a different viewpoint on the findings (Hair et al., 2021). It was suggested that the stronger the association between two variables, the greater the effect size. According to Cohen (1988), *f*^2^ values are interpreted as follows: small effect (≥ 0.02 and < 0.15), medium effect (≥ 0.15 and < 0.35), and significant effect (≥ 0.35) (Hair et al., 2021). Except for the internet technology element, all impact sizes are minor, as shown in Table 9. The endogenous variable was influenced to some extent by each predictor (Figure 4).

**Table 9:** Effect sizes (*f*^2^).

Constructs	*f^2^*	Magnitude
PE	0.057	Small
EE	0.020	Small
SI	0.031	Small
FC	0.037	Small
IT	0.013	No effect
SQ	0.021	Small
TSF	0.008	Small
TSE	0.006	Small
EA	0.025	Small
PI	0.030	Small
PE	0.057	Small

### 4.8 Artificial neural network analysis

**Figure 5:**
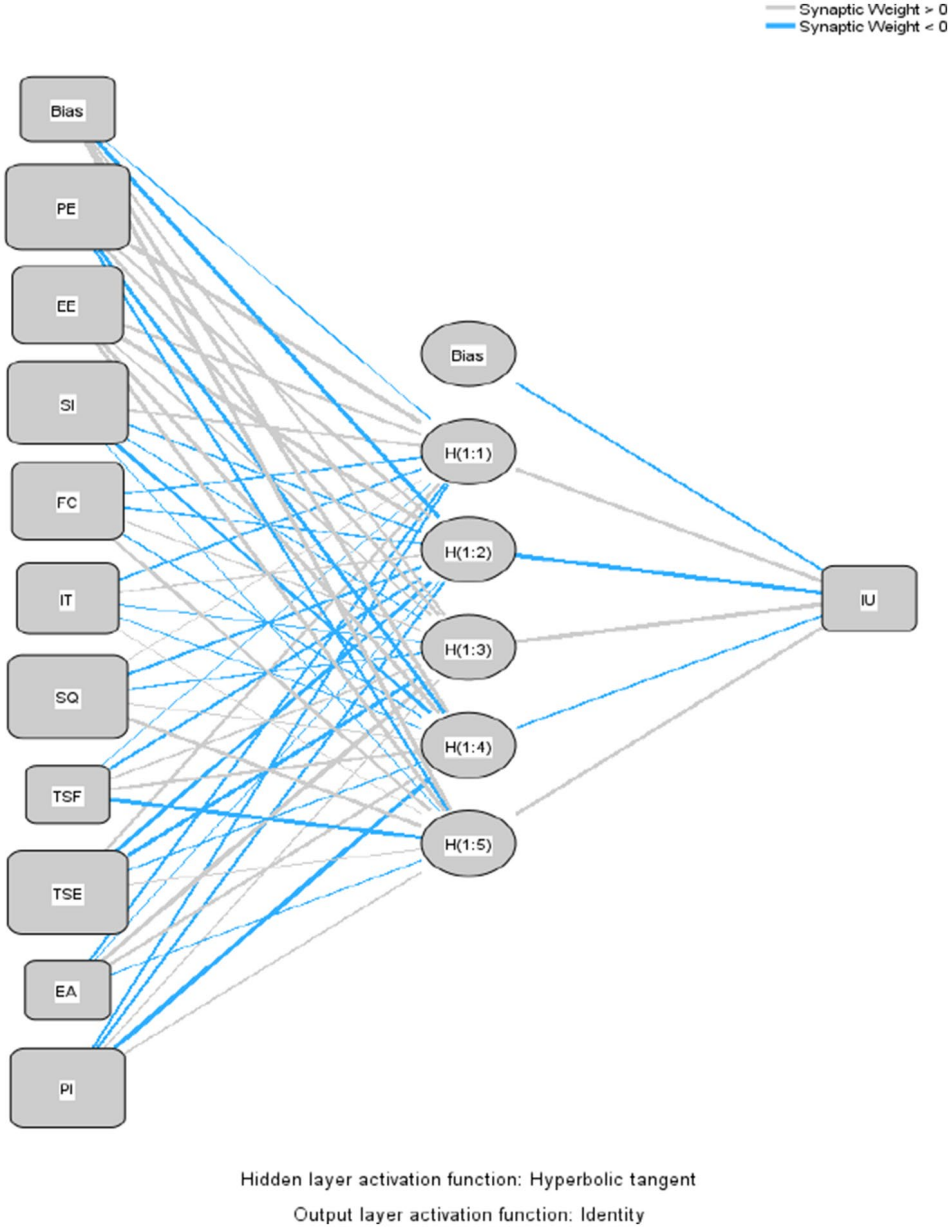
ANN model.

**Figure 6:**
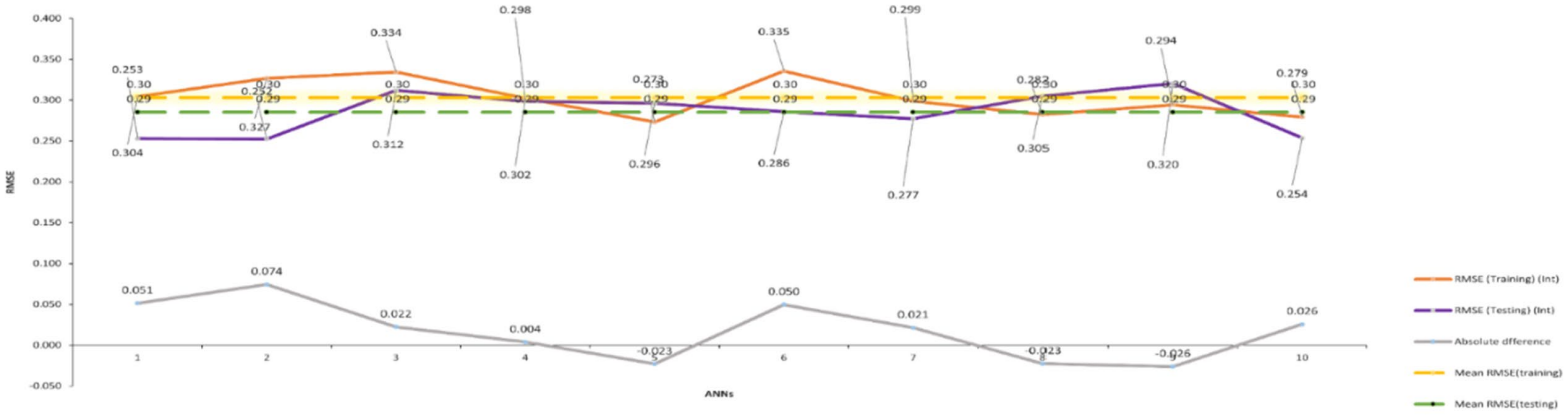
RMSEs for training and testing data, across ANN iteration.

The independent construct obtained from the PLS-SEM result was used in the ANN analysis, which was carried out using SPSS. For every output neuron node, a two-layer deep method was used (Salloum et al., 2020; Lee et al., 2020). A tenfold cross-validation procedure (*r* = 80:20) was used to prevent the ANN model from overfitting (Sharma and Sharma, 2019). Figure 3 illustrates this study model, which was broken down into a single ANN model (Figure 5). The Root Mean Square Error (RMSE) was used to gage prediction accuracy. As a gage of prediction accuracy, RMSE was calculated (Leong et al., 2015). In Table 10, the RMSE of training and testing data were close to each other, indicating an acceptable range to qualify the ANN model from error in Figure 6 (Mishra et al., 2024). ANN number 2 had the highest prediction accuracy due to the lowest RMSE (0.252). Therefore, Figure 6 represented the structure of ANN number 2. The ANN model proved to be highly reliable for determining the relationship between the predictor and output (Liébana-Cabanillas et al., 2018; Karkonasasi et al., 2018; Khan et al., 2023).

**Table 10:** RMSE values.

Network	RMSE (training)	RMSE (testing)	Absolute difference	Mean RMSE (training)	Mean RMSE (testing)
ANN 1	0.304	0.253	0.051	0.300	0.290
ANN 2	0.327	0.252	0.074	0.300	0.290
ANN 3	0.334	0.312	0.022	0.300	0.290
ANN 4	0.302	0.298	0.004	0.300	0.290
ANN 5	0.273	0.296	−0.023	0.300	0.290
ANN 6	0.335	0.286	0.050	0.300	0.290
ANN 7	0.299	0.277	0.021	0.300	0.290
ANN 8	0.282	0.305	−0.023	0.300	0.290
ANN 9	0.294	0.320	−0.026	0.300	0.290
ANN 10	0.279	0.254	0.026	0.300	0.290
Mean	0.30	0.29	0.02	0.300	0.290
Std Dev	0.02	0.03	0.03	0.300	0.290

ANNs, Artificial Neural Networks; RMSEs, The Root Mean Square of Error.

### 4.9 Sensitivity analysis

Sensitivity analysis was performed to assess the normalized significance of predictors. The average relative importance of the 10 neural networks was calculated. Table 11 showed the normalized importance ranged from 65 to 100%. The most significant predictors were performance expectancy (100%), followed by personal innovativeness (95%), social influence (93%), effort expectancy (92%), system quality (87%), facilitating conditions (83%), technology self-efficacy (80%), employee awareness (69%), time-saving features (69%), and internet technology feature (65%).

**Table 11:** Sensitivity analysis of input features.

Network	PE	EE	SI	FC	IT	SQ	TSF	TSE	EA	PI
ANN 1	0.65	1.00	0.99	0.68	0.52	0.68	0.81	0.63	0.86	0.85
ANN 2	1.00	0.78	0.94	0.80	0.63	0.93	0.32	0.94	0.35	0.84
ANN 3	1.00	0.72	0.35	0.77	0.46	0.55	0.48	0.55	0.55	0.42
ANN 4	0.73	0.97	1.00	0.92	0.50	0.69	0.56	0.63	0.77	0.91
ANN 5	0.66	0.33	0.43	0.66	0.41	0.61	0.29	0.52	0.32	1.00
ANN 6	0.11	0.64	0.58	0.72	0.45	1.00	0.35	0.68	0.85	0.98
ANN 7	0.90	0.50	0.59	0.39	0.43	0.40	1.00	0.35	0.34	0.79
ANN 8	0.60	0.58	1.00	0.86	0.58	0.60	0.67	0.66	0.47	0.71
ANN 9	1.00	0.75	0.63	0.23	0.32	0.51	0.15	0.70	0.27	0.28
ANN 10	1.00	0.73	0.57	0.34	0.65	0.68	0.66	0.46	0.48	0.50
Average importance normalized importance %	100%	92%	93%	83%	65%	87%	69%	80%	69%	95%

The accuracy of the ANN model (79.80%) in predicting the account executive’s desire to adopt AI was compared to that of the PLS-SEM model (80.40%). The nonlinear links between the constructs were identified using an ANN’s deep learning implementation, which contributed to the discrepancy in accuracy (Almarzouqi et al., 2022a).

The variables “performance expectancy, facilitating condition, personal innovativeness, employee awareness, effort expectancy, social influence, system quality, time-saving feature, technological self-efficacy, and internet technology feature” were ranked in order of relative importance in Table 12‘s PLS-SEM results. But according to the ANN model, the most significant predictors of the intention to use AI were “performance expectancy, personal innovativeness, social influence, effort expectancy, system quality, facilitating condition, technological self-efficacy, employee awareness, time-saving feature, and internet technology feature”.

**Table 12:** Summary of ranking importance.

Constructs	PLS-SEM	ANN sensitively
PE	1	1
EE	5	4
SI	6	3
FC	2	6
IT	10	9
SQ	7	5
TSF	8	8
TSE	9	7
EA	4	8
PI	3	2

## 5 Discussion

Artificial intelligence has the potential to help account executives reduce their workload, automating tasks such as data entry, invoice processing, and decision-making (Ng and Alarcon, 2020). Research is thus required to determine account executives’ intentions to employ AI in SMEs, through the expansion of UTAUT, as well as technical and personal characteristics. The results of the validity and reliability tests show a strong basis for further study and measurement utilizing the same model. Three hypotheses did not support the research model out of 10 hypotheses identified for this study.

### 5.1 Relationship between UTAUT on intention to use artificial intelligence among SME account executives

The initial emphasis of hypothesis formation is based on four primary UTAUT constructs: performance expectancy, effort expectancy, social influence, and enabling factors. The hypotheses are divided into H1, H2, H3, and H4, which investigate the intentions of SME account executives to utilize AI. These ideas suggest that UTAUT and the propensity of SME account executives to embrace AI are significantly correlated. All the UTAUT basic components have a positive and substantial correlation with SME account executives’ desire to use AI.

#### 5.1.1 Relationship of performance expectancy on intention to use artificial intelligence among SME account executives

In line with the results of earlier studies, account executives’ intention is significantly positively impacted by performance expectancy (Jameel et al., 2023; Bouteraa et al., 2024; Nascimento and Meirelles, 2022). According to numerous researchers, the intention to use AI in industries such as travel and tourism, healthcare (Sitthipon et al., 2022), and life coaching is influenced by performance expectancy (Terblanche and Kidd, 2022; Sitthipon et al., 2022; Melián-González et al., 2021; Dávid and Dadkhah, 2023). Females make up 65.20 per cent of the responses, representing women in the accounting industry. Consequently, the usefulness and efficacy of AI are of greater significance to women. The 25–34 age range, which is represented by 43.20% of respondents, is known for having a greater level of technology involvement and being receptive to new developments. The use of technology among younger populations is more likely to boost productivity and efficiency at work. According to Jameel et al. (2022), performance expectation also contributes to improved work performance (Naji et al., 2022). Employees are more willing to study technology if they feel that it will help them accomplish tasks efficiently and effectively. A higher level of performance expectancy increases the intention to use AI (Bouteraa et al., 2024). Account executives increasingly adopt AI due to its significant contributions to their work performance. The technology supports decision-making processes and automates repetitive procedures, among other advantages. This result helps policymakers, SMEs, and technology developers recognize the importance of performance benefits in raising awareness of the potential applications of AI in work settings.

#### 5.1.2 Relationship of effort expectancy on intention to use artificial intelligence among SME account executives

Consistently, effort expectation also has a favorable and substantial impact on account executives’ intentions (Nascimento and Meirelles, 2022; Akbar et al., 2023). Newer technologies in an organization are more straightforward to employ if users possess more technical innovation skills (Ikumoro and Jawad, 2019). The ambition to employ the technology is encouraged by its simplicity (Utomo et al., 2021). Employees are inclined to adopt AI because it simplifies their work duties (Akbar et al., 2023). According to Nascimento and Meirelles (2022), the implementation of AI is hindered by its complexity. If integrating AI into their operations is straightforward, employees are more likely to utilize it. The desire of employees to utilize AI increases when they receive training on its use and the user interface is simplified. The ambition to apply AI decreases with increasing complexity. The desire to embrace technology is increased when employees need less effort to understand and utilize a new system. Because SMEs have limited time and resources, employees’ intentions are influenced by how simple it is to integrate and use new technology (Kwarteng et al., 2024). Younger respondents (25–34 years old) in this poll indicated that 43.20% are more inclined to use AI in their employment. Workers over 45 may have difficulties since they are less accustomed to the technology, which will make AI seem more complicated. This indicates that younger workers embrace AI faster than elderly workers, who could require more training or more user-friendly interfaces.

#### 5.1.3 Relationship of social influence on intention to use artificial intelligence among SME account executives

Results of earlier research show that social influence had a significant impact on account executives’ intentions (Nascimento and Meirelles, 2022; Kandoth and Shekhar, 2022). Even if employees find it challenging to use, if influenced by friends or family, they may still try to use it. The industry-wide adoption of technologies will also influence it. Companies used AI when it became a standard practice within an industry (Nascimento and Meirelles, 2022). In a collective society, individuals are often influenced by their peers, colleagues, friends, and family. High-status individuals are more likely to counsel low-status individuals on their plan to employ AI. The likelihood of utilizing AI increases with the influence of a friend (Kandoth and Shekhar, 2022). Therefore, family, friends, and lovers influence users positively or negatively in their adoption of technology in SMEs within their social environment (Aziz et al., 2022). Younger participants are influenced by social media (Menon and Shilpa, 2023). 43.20% of those surveyed are between the ages of 25 and 34, who are more likely to adopt new technology if encouraged by their peers or social media. The sharing or influence by peers or family increases the intention to use AI (Wang et al., 2025). When their social counterparts utilize AI, account executives are also more likely to adopt it.

#### 5.1.4 Relationship of facilitating conditions on intention to use artificial intelligence among SME account executives

If the existing technological infrastructure is easy to use and promotes the use of AI, employees are more likely to embrace it (Jameel et al., 2023). Organizations can enhance workers’ desire to utilize AI by providing them with the necessary resources and support (Nascimento and Meirelles, 2022). Facilitating environments promote the utilization of technology and reduce opposition. This facilitation relates to training and technical assistance. Sufficient enabling circumstances facilitate the reduction of difficulties associated with introducing new technologies (Kwarteng et al., 2024). Like earlier research, facilitating environments have a good and substantial impact on account executives’ intentions (Jameel et al., 2023; Kwarteng et al., 2024). SMEs often lack resources, but if provided with proper technological infrastructure and encouraged to use AI in the workplace, account executives are willing to adopt it. 54.20% of employees in the higher income group (RM2001-RM5000) might have better access to training and resources, which increases the intention to use AI. However, the 4.80% lower-income group (below RM2000) may face barriers to using AI due to limited tools or support systems.

### 5.2 Relationship between technological attributes and intention to use artificial intelligence among SME account executives

The second topic of the study’s hypothesis is extending the new determinant of UTAUT to the desire of SMEs’ account executives to employ AI. It is what H5, H6, and H7 present. Except for H5 and H7, this investigation demonstrated that H6 is supported.

#### 5.2.1 Relationship between internet technology features on intention to use artificial intelligence among SME account executives

Internet Technology (IT) features show a negative relationship with the intention to use AI among SME account executives (Bin-Nashwan and Al-Daihani, 2021). This may be because AI is still new, and concerns about security, trustworthiness, and privacy matters exist. Furthermore, the lack of adequate digital literacy and necessary skills exacerbates this issue. These results align with earlier research (Bouteraa et al., 2024). Age-wise, the most considerable percentage of responders (43.20%) was in the 25–34 age group, followed by those in the 18–24 years old age group (32.60%). Those age groups are more open to technology innovation; therefore, many respondents in this age range have limited knowledge of the technology, especially as accounts are time-sensitive and confidential jobs. This also reflects that female respondents express heightened concerns about the security of AI systems and the potential for data misuse of personal information.

#### 5.2.2 Relationship between system quality features on intention to use artificial intelligence among SME account executives

There was a positive and substantial correlation between system quality and the intention of SME account executives to employ AI. When people perceive the service quality of innovative technology more positively, they are more likely to share it with others (Nan et al., 2024). Better system quality leads to increased system usage (Anggreni et al., 2020). System quality is essential, especially for AI adoption, because account executives often seek tools to help them perform tasks efficiently and effectively. Female respondents (65.20%) are more concerned about the reliability and availability of the systems that ensure fast response times and are available on time. Those aged 18–31 are more likely to expect high-tech systems that can function seamlessly. Furthermore, respondents with higher incomes (RM2001-RM5000 and above RM5000) are likely to have better system quality, meaning they expect faster response times and greater system availability.

#### 5.2.3 Relationship between time-saving feature on intention to use artificial intelligence among SME account executives

The time-saving feature showed a negligible relationship to the use of AI by SME account executives. Account executives may not immediately perceive the benefits of time savings due to the novelty of the new technology and the need to overcome the learning curve associated with its use. When it comes to their desire to employ AI, users are more concerned about the technology’s usability. This outcome is consistent with earlier research (Zimu, 2022). Younger respondents, especially those in the 18–34 age group, are more open to exploring AI tools, but do not immediately recognize the benefits of time savings. This group often focuses more on integrating technology into their daily work tasks to improve workflow efficiency, rather than achieving immediate time-saving outcomes.

### 5.3 Relationship between individual attributes and intention to use artificial intelligence among SME account executives

The third topic of the hypothesis is extending the new determinant of UTAUT to the desire of SMEs’ account executives to employ AI. H8, H9, and H10 present it. This study proves that H9 and H10 are supported, except for H8.

#### 5.3.1 Relationship between technological self-efficacy and intention to use artificial intelligence among SME account executives

Technological self-efficacy showed a negligible relationship to the use of AI by SME account executives. SMEs often operate with limited resources, and employees may not have sufficient training opportunities to develop strong technology self-efficacy, particularly in the context of AI (Sullivan-Taylor and Branicki, 2011). In this study, younger respondents aged between 18 and 24 are more open to open innovation technology. However, if the respondents do not receive proper training and support, their confidence in using AI may be hindered. Older employees may find AI more challenging due to their lesser familiarity with the technology and fewer opportunities for upskilling, especially in the SME industry.

#### 5.3.2 Relationship between employee awareness of intention to use artificial intelligence among SME account executives

The aim of SME account executives to apply AI was positively and significantly correlated with employee awareness (Bouteraa et al., 2024; Flavián and Casaló, 2021). It highlights the importance of informing people that AI is user-friendly and beneficial for financial management. Customers’ knowledge of AI may be raised by offering them user-friendly apps and a straightforward interface (Flavián et al., 2021). By ensuring that consumers are aware of the advantages and capabilities of AI, awareness may help lower resistance to change and improve mobile payment systems. Companies provided employees with regular and clear communication about the new technology. The training program will introduce employees to the new systems (Flavián et al., 2020). In this study, respondents aged between 25 and 34 (43.20%) and female respondents showed a higher awareness of AI, which may be due to their participation in training or their increased attentiveness to recent technological advancements worldwide. Account executives will be more inclined to employ AI if they become more knowledgeable about it.

#### 5.3.3 Relationship between personal innovativeness and intention to use artificial intelligence among SME account executives

Personal innovativeness is characterized by an individual’s inclination to embrace new and unfamiliar activities (Ciftci et al., 2021). Individuals with a high degree of personal innovativeness will have a strong perspective on advanced technology (Agarwal and Prasad, 1998). The results indicate that personal innovativeness has a positive and significant relationship with the intention to use AI among SME account executives. Early users of new technology will primarily be innovative people. This evidence confirms earlier research reported by Abbasi et al. (2021), Kandoth and Shekhar, 2022, and Bouteraa et al. (2024). Individuals who exhibit high levels of inventiveness overcome the dangers by utilizing new technologies and adopting new ideas or technologies. The technology of mobile payment systems will be used more frequently by those who are more receptive to innovation. They are willing to alter their routines because they recognize the benefits of the new technology (Naji et al., 2025b). An individual’s attitude toward innovation and intention toward technology will be more favorable if they possess a higher degree of personal innovativeness (Kandoth and Shekhar, 2022). In this study, the majority of respondents are female (65.20%), indicating that females have more innovativeness than males. The income level for RM2001-RM5000 (54.20%) and above RM5000 demonstrates a higher level of innovativeness, likely due to the financial resources that enable them to explore and adopt new technologies, compared to individuals with an income below RM2000.

### 5.4 Implication

#### 5.4.1 Theoretical implication

This study makes notable theoretical contributions to the fields of artificial intelligence and technology adoption. Building upon the foundational elements of the Unified Theory of Acceptance and Use of Technology (UTAUT), namely Performance Expectancy, Effort Expectancy, Social Influence, and Facilitating Conditions, the research extends the model by integrating additional constructs that reflect both technological and individual characteristics. These include Internet Technology (IT) features, system quality, time-saving features, technological self-efficacy, employee awareness, and personal innovativeness. By incorporating these dimensions, the study offers a more comprehensive framework for understanding the intention to adopt AI among SME account executives, particularly in environments where resource limitations and digital readiness vary significantly.

Two significant contributions of current knowledge. Firstly, it demonstrates how the UTAUT paradigm can be tailored to a new situation, specifically the application of read-write AI technology in SMEs. These extra constructs tackle the potential and difficulties of adopting AI, especially in settings with limited resources, such as SMEs. For instance, factors such as technological self-efficacy highlight the importance of users’ confidence in interacting with AI technologies. At the same time, attributes like system quality and time-saving features capture the usability and efficiency aspects that are vital in practical adoption scenarios. In addition to enhancing the theoretical foundation, this expansion of UTAUT offers a customized understanding of AI adoption in SMEs, where dynamics differ significantly from those in larger organizations. Second, add literature on technology adoption by focusing on SMEs and their account executives. While most prior studies have focused on large enterprises or general populations, this study fills a critical gap by examining a key professional group within SMEs. The insights generated offer a context-specific perspective that enhances the relevance and applicability of existing theories. Additionally, focuses on adopting AI in accounting processes, increasing domain-specific knowledge, and contributing to AI research and SME literature.

This study’s methodological approach, which blends Artificial Neural Network (ANN) analysis with Partial Least Squares Structural Equation Modeling (PLS-SEM), is one of its unique features (Quan et al., 2023; Nguyen et al., 2023). This dual-method approach strengthens the theoretical implications by providing robust, multi-dimensional insights into the factors shaping AI adoption intentions. While PLS-SEM identifies and validates the relationships among constructs, ANN offers a nonlinear, predictive analysis, further enhancing the explanatory power of the findings. This combination not only reinforces the reliability of the results but also sets a precedent for future research seeking to adopt a multi-method approach in technology adoption studies.

Using the UTAUT framework, this study advances knowledge of AI adoption in SMEs and highlights the importance of creative methodological approaches in theoretical research. Future research examining the interaction of organizational, technological, and individual aspects in AI adoption will have a solid basis thanks to their contributions.

#### 5.4.2 Practical implication

This research offers practical insights for SME accounting departments, policymakers, and industry stakeholders seeking to adopt AI. AI presents transformative opportunities for SMEs, enabling them to enhance decision-making, reduce expenses, and improve operational efficiency. Artificial intelligence (AI) reduces human error. It frees staff members to focus on higher-value, strategic tasks by automating labor-intensive processes, such as bookkeeping, invoicing, tax calculations, and fraud detection. Furthermore, AI’s real-time analysis enables SMEs to predict financial trends, optimize cash flow, and enhance long-term planning, making them more agile and competitive in a rapidly evolving market.

SMEs must address employee resistance to and concerns about AI to achieve effective adoption. Positioning AI as a tool to supplement their responsibilities is essential, as employees may view it as a threat to their job security. Organizations can implement awareness campaigns, interactive workshops, on-the-job training, and user-friendly tutorials to facilitate the adoption of AI technologies. These approaches can highlight AI’s advantages, such as improved task accuracy, reduced costs, and enhanced decision-making capabilities. To encourage confidence and participation, SMEs should establish a safe learning environment where staff members can experiment with AI tools without worrying about making mistakes. Furthermore, the learning curve will be reduced, and integration will proceed more smoothly if AI systems are safe, simple to use, and interoperable with existing technology.

This study provides valuable insights for legislators seeking to develop policies that promote the adoption of AI. Governments can alleviate the financial burden on small and medium-sized businesses by offering financial support in the form of grants, subsidies, or tax incentives. This will enable these businesses to invest in AI technology. Furthermore, clear regulatory frameworks addressing critical issues such as data privacy, ethics, and liability are essential to guiding SMEs through the complexities of AI implementation. By implementing these guidelines, governments can enhance confidence and ensure compliance with legal requirements, thereby creating a safe and open environment for AI integration. Another crucial area requiring government involvement is enhancing workforce skills. Policymakers can support AI-centric training initiatives and collaborate with educational institutions to provide employees with the essential skills for effective AI utilization. Joint efforts between governmental bodies and industry experts can expedite the development of scalable, readily available AI solutions tailored to meet the needs of small businesses.

It also highlights the importance of industry collaboration. SMEs can partner with AI vendors, technology providers, and academic institutions to co-develop AI solutions that address their unique operational challenges. Sharing best practices and participating in knowledge-sharing initiatives can further enhance SMEs’ ability to implement AI successfully.

In conclusion, practical implications provide SMEs and policymakers with a comprehensive roadmap for implementing AI in a meaningful and sustainable manner. By addressing the specific challenges that SMEs encounter, including resource constraints and employee resistance, this research proposes effective strategies to facilitate the adoption of AI. These findings will enable SMEs to maintain their market position, enhance operational effectiveness, and thrive in an increasingly technology-driven business landscape.

### 5.5 Limitation

Despite the compelling results, certain constraints apply. A cross-sectional design was adopted over a brief period, which may result in a less comprehensive analysis. This is because contextual and temporal variations could yield different outcomes, necessitating caution when drawing broad conclusions. Furthermore, the research’s narrow focus on the SME sector within a single country (Malaysia) might restrict its applicability to other industries and nations. Diverse results might result from cultural, economic, and regulatory variations. This may impact the Validity of constructs. The study assumes that participants have some knowledge of AI technologies. However, different levels of digital competence might affect the results.

### 5.6 Future direction

PLS-SEM and ANN offer predictive insights. It is necessary to consider a similar research study incorporating a longitudinal design with ample time to obtain more precise results. This provides insights into the nature of adopting technology, capturing changes in employee attitudes, organizational readiness, and technological advancement. Future studies could incorporate objective measurements, such as AI adoption or performance metrics, to confirm the results. Results are based on 273 account executives from SMEs in Malaysia, which limits their generalizability to other regions and industries. To improve external Validity, more study is advised to broaden the geographic focus and incorporate a variety of sectors. A hybrid approach will be adopted in future research to gain a deeper understanding of the applications of AI. Interviews and other qualitative techniques can offer a more profound comprehension of the interviewees, including their emotions and behaviors. Future research can explore the application of various AI in various departments, such as marketing, operations, or human resources. Stratified sampling based on technology proficiency can help better understand how factors influence intention to use AI. SMEs can enhance their adoption of AI by developing targeted education and training programs. Cooperation between SMEs and outside AI specialists may increase knowledge, resources, and best practices for integrating AI.

## 6 Conclusion

The study’s factors (performance expectancy, effort expectancy, social influence, facilitating conditions, system quality, employee awareness, and personal innovativeness) show a strong positive correlation with SME account executives’ intention to use AI. However, the use of AI by SME account executives was not significantly correlated with internet technology characteristics (negative and significant), technological self-efficacy, or time-saving features. The results were further supported using Artificial Neural Network (ANN) research, which also offered new insights into the relative importance of these predictors. The ANN findings substantiated that performance expectancy was the most crucial factor (100% normalized importance), with personal innovativeness (95%), social influence (93%), and effort expectancy (92%) following in order of importance. The ANN’s capacity for nonlinear modeling also uncovered subtle relationships not detected by PLS-SEM, enhancing the study’s credibility. The model demonstrated high predictive accuracy, achieving an R^2^ of 79.80%, which corresponded to the PLS-SEM model’s R^2^ of 80.40%. According to this study, the goal of SME account executives to employ AI is better understood. The research’s implications align with those of SMEs’ account executives. This study contributes to the ongoing efforts to enhance the operations of SMEs in Malaysia by examining the desire of account executives to adopt AI in SMEs. The results highlight the importance of modifying tactics to address factors in the goal of utilizing AI. Stakeholders and policymakers may use this information to develop requirements or regulations that encourage SMEs to adopt AI. Additionally, the application of cutting-edge approaches to better understand user behavior and decision-making processes is highlighted by the use of sophisticated analytical tools, such as artificial neural networks (ANN).

## Data Availability

The raw data supporting the conclusions of this article will be made available by the authors, without undue reservation.
