# Supplementary material for: Intention to use artificial intelligence among SME account executives
Source: Front Artif Intell. 2026 Feb 6;9:1701133. doi: 10.3389/frai.2026.1701133 (PMC12920514; doi:10.3389/frai.2026.1701133)
Supplement: Supplementary file 1 [file Data_Sheet_1.docx]

**Appendix**

**APPENDICES**

Appendix I

*Selected studies of intention on Artificial Intelligence in SMEs*

| **Author (Year)** | **Objective** | **Country of survey** | **Theory** | **Findings** |
| --- | --- | --- | --- | --- |
| [1] | To identify factor for adoption of artificial intelligence in SMES. | Jordan | Technology–organisation–environment (TOE) | IT Knowledge, IT Infrastructure, Managerial Commitment, Training Initiative and Reward System Are Significant Impact of adopt artificial intelligence in SMEs |
| [2] | To study factors that most influential the adoption of artificial intelligence in SMEs | Saudi Arabi | Technology–organisation–environment (TOE) | Human capital is the most crucial factor that influences SMEs to use artificial intelligence. |
| [3] | To identify the factors that influence the adoption of artificial intelligence in small companies in Brazil | Brazil | Unified Theory of Acceptance and Use of Technology (UTAUT) | Nine factors (performance expectancy, business model, effort expectancy, self-efficacy, trust, business compatibility, social influence, trial ability and technical support) are the factors that influence the adoption of artificial intelligence in SMEs |
| [4] | To overcome the limitation of previous studies and identify which factors influence the adoption of artificial intelligence in SMEs. | Portugal | Multi-criteria decision-support system | Human resource, IT infrastructure, know-how and knowledge; organizational policies and management; and leadership will influence adoption of artificial intelligence in SMEs |
| [5] | To determine the factor of adoption of artificial intelligence in SME | Malaysia | Systematic Literature review | Top management commitment and organization readiness have a significant relationship with artificial intelligence adoption.  Competitive pressure, employee adaptability and external support insignificant relation with artificial intelligence adoption in SMEs. |
| [6] | To determine the behavioural intention to use artificial intelligence among managers in SME | Baghdad | Unified Theory of Acceptance and Use of Technology (UTAUT)  Top Management support (TMS) | Performance expectancy, social influence, facilitating conditions, and top management support positively impact managers' intentions to use artificial intelligence in SMEs.  Effort expectancy has an insignificant impact on behavioural intention to use artificial intelligence among managers in SMEs |
| [7] | To examine the factor influence the adoption of artificial intelligence in SMEs | India | Systematic Literature review | Employee adaptability, competitive pressure and external support are significantly influenced adaptation of artificial intelligence in SMEs.  Top management support and organisation readiness are significantly influenced adaptation of artificial intelligence in SMEs. |

Appendix II

Selected studies of intention on technology in SMEs

| **Author (Year)** | **Objective** | **Country of survey** | **Theory** | **Findings** |
| --- | --- | --- | --- | --- |
| [8] | To examine the factor influence Women to adopt technology in SMEs | Cambodian | Systematic Literature review | Perceived advantages and commercial potential will influence the adaptation of technology. |
| [9] | To identify the factor influence the adoption of digitalization technology in SMEs | European | Unified Theory of Acceptance and Use of Technology (UTAUT) | Performance expectancy, facilitating conditions, competitive pressure are significantly after the adopt of digitalization technology in SMEs. |
| [10] | To identify the intention of adopt information technology in SMEs. | Indonesia | Technology acceptance model (TAM) | Pressure of business competition are influence on the perceived usefulness by SME.  Perceived ease of use and perceived usefulness are significant relationship to attitude of SME to information technology adoption.  Attitude SME Information technology adoption will influence owner to adopt it. |
| [11] | To identify the factor influence of technology in SMEs . | Indonesia | Technology acceptance model (TAM) | Perceived usefulness, perceived ease of use significant relationship on attitude. Attitude has significant relationship to behaver intention to use technology. |
| [12] | To identify the intention to use technology in SMEs . | Denpasar | Technology acceptance model (TAM) | Perceived ease of use is positive effect to perceived usefulness and intention to use technology.  Perceived Risk are positive effect intention to use technology.  Perceived usefulness no effect intention to use technology.  Intention to use technology positive effect on Usage Behaviour.  Intention to use technology can mediate the effect on perceived ease of use and perceived usefulness on usage behaviour but cannot media the perceived risk to usage behaviour.  Perceived usefulness cannot mediate the effect of perceived ease of use and intention to use technology. |

| Appendix II  *Adapted Measurement Items for Questionnaire* | | | | |
| --- | --- | --- | --- | --- |
| **Variable** | **No** | **Original Items** | **Adapted Items** | **Source** |
| Performance Expectancy | PE1 | Using the PeduliLindungi application can increase productivity when carrying out  activities | Using Artificial Intelligence can enhance the productivity during work. | (Akbar et al., 2023) |
|  | PE2 | Using the PeduliLindungi application can make it easier to do activities and not be convoluted | Using Artificial Intelligence can simplify the execution of task and reduce the complexity of task. |  |
|  | PE3 | Using the PeduliLindungi application can speed up activities | Artificial Intelligence can help to speed up the work. |  |
|  | PE4 | Using the PeduliLindungi Application will produce quality output | Artificial Intelligence will improve the quality of work output. |  |
|  | PE5 | Using the PeduliLindungi Application  makes activities easier than before using the PeduliLindungi Application | Artificial Intelligence make task easier compare to before. |  |
| Effort Expectancy | EE1 | The features in the PeduliLindungi application are simple so that it is easy to understand | Feature of Artificial Intelligence are straightforward and can be easy to understand it. | (Akbar et al., 2023) |
|  | EE2 | Features in the PeduliLindungi application are simply easy to learn | Artificial Intelligence are simple to learn. |  |
|  | EE3 | The features in the PeduliLindungi application are simple so it is easy to run | Artificial Intelligence are easy to use and operate. |  |
|  | EE4 | The interaction between users and the PeduliLindungi application is clear so it is very easy to understand | Using the  Artificial Intelligence is clear  and easy to understand. |  |
| Social influence | SI1 | I use the PeduliLindugi app because my friend use | I use Artificial Intelligence due to my friend use it. | (Akbar et al., 2023) |
|  | SI2 | I use the PeduliLindugi app because my family uses | I use Artificial Intelligence due to my family use it. |  |
|  | SI3 | I use the PeduliLindugi application because of the applicable policies | I use Artificial Intelligence due to company policies. |  |
|  | SI4 | I use the PeduliLindugi application because of the influence of the organization | I use Artificial Intelligence due to influence of my organization. |  |
|  | SI5 | I use the PeduliLindungi application because of the influence of social media | I use Artificial Intelligence due to influence of social media. |  |
|  | SI6 | I use the PeduliLindugi application because my surroundings have used the PeduliLindungi application a lot | I use Artificial Intelligence due to my  surroundings have use it a lot. |  |
|  | SI7 | Using the PeduliLindungi application can improve my self-image | I use Artificial Intelligence to improve my self-image. |  |
| Facilitating condition | FC1 | I have the facility to access the PeduliLindungi application (smartphone) | I have the necessary facility to access Artificial Intelligence. | (Akbar et al., 2023) |
|  | FC2 | I have enough ability and knowledge to use the PeduliLindungi application | I have enough ability and knowledge to use Artificial Intelligence. |  |
|  | FC3 | Some experts can help me when I have trouble using the PeduliLindungi application | Some Experts are available to help me when I have trouble using Artificial Intelligence. |  |
|  | FC4 | I feel that the PeduliLindungi application is a system that can work accordingly | Artificial Intelligence can help work effectively. |  |
| Internet Technology Feature | IT1 | I am concerned about the Internet security | I am concerned the internet security when using Artificial Intelligence. | (Sura et al., 2017) |
|  | IT2 | I am concerned about the online transaction reliability | I am concerned the online transaction reliability when using Artificial Intelligence. |  |
|  | IT3 | I am concerned about the Internet trustworthiness | I am concerned the trustworthiness when using Artificial Intelligence. |  |
|  | IT4 | I am concerned about Internet privacy | I am concerned the online privacy when using Artificial Intelligence. |  |
|  | IT5 | I am concerned about Internet effectiveness^∗^ | I am concerned the effectiveness of internet when using Artificial Intelligence. |  |
| System Quality | SQ1 | Fintech services keep error-free transaction | Artificial Intelligence can reduce the error. | (Bouteraa, Chekima, et al., 2023) |
|  | SQ2 | Fintech services have a fast transaction processing time | Artificial Intelligence are provide fast response time. |  |
|  | SQ3 | Fintech services are reliable | Artificial Intelligence are reliable. |  |
|  | SQ4 | Fintech services can be used at anytime | Artificial Intelligence can be use in any times. |  |
|  | SQ5 | Fintech services have good functionality relevant to my transaction | Artificial Intelligence are function well to my work. |  |
| Time Saving Feature | TSF1 | Using green banking provides me access to bank anytime-anywhere | Artificial Intelligence can save my time. | (Iqbal et al., 2018) |
|  | TSF2 | Using green banking reduces the waiting time for my banking transactions | Artificial Intelligence can reduce my routine task. |  |
|  | TSF3 | Using green banking provides me banking information in a timely fashion | Artificial Intelligence can provide me the information. |  |
|  | TSF4 | Using green banking makes transactions faster by reducing human interventions | Artificial Intelligence can reduce the manual human work. |  |
| Technology Self Efficacy | TSE1 | I feel confident in my ability to use social media to have meaningful interactions. | I am confident in my ability to use Artificial Intelligence during work. | (Saville & Foster, 2021) |
|  | TSE2 | I feel confident in my ability to use technology for entertainment. | I am confident in my ability to use Artificial Intelligence automating to replace my routine accounting task. |  |
|  | TSE3 | I feel confident in my ability to use Internet tools to conduct research and find trustworthy articles on a topic. | I am confident in my ability to use Artificial Intelligence to generate financial report. |  |
|  | TSE4 | I feel confident in my ability to use technology to create an engaging presentation. | I am confident in my ability to use Artificial Intelligence into company existing accounting software. |  |
|  | TSE5 | I feel confident in my ability to use new applications on my smartphone or tablet. | I am confident in my ability to use Artificial Intelligence on my smartphone or tablet. |  |
| Employee Awareness | EA1 | I am aware of the existence of fintech services | I am aware for the Artificial Intelligence. | (Bouteraa, Chekima, et al., 2023) |
|  | EA2 | I am aware of the concept of fintech services | I am aware for the concept of Artificial Intelligence. |  |
|  | EA3 | I know the purpose of fintech services | I am aware for the purpose of using Artificial Intelligence in workplace. |  |
|  | EA4 | I know the benefits of using fintech services | I agree with the benefit that bring by Artificial Intelligence. |  |
|  | EA5 | In general, I have enough information about fintech services | I feel I know all the usage of Artificial Intelligence. |  |
| Personal Innovativeness | PI1 | If I hear about new technology, I look for ways to experiment with it | I will try a new technology when I hear it. | (Bouteraa, Chekima, et al., 2023) |
|  | PI2 | I am usually the first to try new information technologies Among my peers | I will be the first user to try the new technologies among my peers. |  |
|  | PI3 | In general, I am not hesitant to try out new information technologies | I am not hesitant to experiment with new technology. |  |
|  | PI4 | I like to experiment with new information technologies | I enjoy exploring the new technology. |  |
| Intention to use | IU1 | I intend to use the system in the next months. | I intend to continue using Artificial Intelligence software in my in the future. | (Venkatesh et al., 2003) |
|  | IU2 | I predict I would use the system in the next months | I will always try to use Artificial Intelligence in my daily work. |  |
|  | IU3 | I plan to use the system in the next months. | I plan to use the Artificial Intelligence frequently. |  |

Appendix IV

*Survey Questionnaire*

**Screening Question**

## **Section 1: Company Classification**

1. Is the company you work for classified under the SME categories mentioned below?

☐ Yes (if yes, proceed to the next question)

☐ No (if no, do not continue. Thank you for your time)

## **Section 2: Current Job Role**

1. Are you currently working as an Account Executive?

☐ Yes (if yes, proceed to the next question)

☐ No (if no, do not continue. Thank you for your time)

## **Section 3: Work Experience in SMEs**

1. Do you have a minimum of 1 year of working experience within SMEs?

☐ Yes (if yes, proceed to the next question)

☐ No (if no, do not continue. Thank you for your time)

## **Section 4: Familiarity with Artificial Intelligence Technology**

1. Do you have knowledge related to Artificial Intelligence technology?

☐ Yes (if yes, proceed to the next question)

☐ No (if no, do not continue. Thank you for your time)

## **Section 5: Use of Artificial Intelligence in Accounting Functions**

1. Have you ever used Artificial Intelligence in your professional role for accounting purposes?

☐ Yes (if yes, do not continue. Thank you for your time)

☐ No (if no, proceed to the next question)

## **Section 6: Demographic Details**

1. Gender:

☐ Male

☐ Female

2. Age:

☐ 18-24

☐ 25-34

☐ 35-44

☐ 45-54

☐ 55 and above

3. Monthly income:

☐ Below RM2000

☐ RM2001-RM5000

☐ Above RM5000

4. Residing region/state:

☐ Johor

☐ Kedah

☐ Kelantan

☐ Melaka

☐ Negeri Sembilan

☐ Pahang

☐ Perak

☐ Perlis

☐ Pulau Pinang

☐ Selangor

☐ Terengganu

☐ Sabah

☐ Sarawak

## **Section B: Attitude Towards Artificial Intelligence**

Instruction: Please read each statement carefully and indicate the answers clearly by choosing the appropriate selection on the scale given below. Kindly select the number that you feel best describes your level of agreement.

1 - Strongly Disagree
2 - Disagree
3 - Neutral
4 - Agree
5 - Strongly Agree

PE1 - Using Artificial Intelligence can enhance productivity during work.
☐ 1 ☐ 2 ☐ 3 ☐ 4 ☐ 5

PE2 - Using Artificial Intelligence can simplify tasks and reduce complexity.
☐ 1 ☐ 2 ☐ 3 ☐ 4 ☐ 5

PE3 - Artificial Intelligence can help to speed up work.
☐ 1 ☐ 2 ☐ 3 ☐ 4 ☐ 5

PE4 - Artificial Intelligence will improve the quality of work output.
☐ 1 ☐ 2 ☐ 3 ☐ 4 ☐ 5

PE5 - Artificial Intelligence makes tasks easier compared to before.
☐ 1 ☐ 2 ☐ 3 ☐ 4 ☐ 5

EE1 - Features of Artificial Intelligence are straightforward and easy to understand.
☐ 1 ☐ 2 ☐ 3 ☐ 4 ☐ 5

EE2 - Artificial Intelligence is simple to learn.
☐ 1 ☐ 2 ☐ 3 ☐ 4 ☐ 5

EE3 - Artificial Intelligence is easy to use and operate.
☐ 1 ☐ 2 ☐ 3 ☐ 4 ☐ 5

EE4 - Using Artificial Intelligence is clear and easy to understand.
☐ 1 ☐ 2 ☐ 3 ☐ 4 ☐ 5

SI1 - I use Artificial Intelligence because my friend uses it.
☐ 1 ☐ 2 ☐ 3 ☐ 4 ☐ 5

SI2 - I use Artificial Intelligence because my family uses it.
☐ 1 ☐ 2 ☐ 3 ☐ 4 ☐ 5

SI3 - I use Artificial Intelligence due to company policies.
☐ 1 ☐ 2 ☐ 3 ☐ 4 ☐ 5

SI4 - I use Artificial Intelligence due to influence from my organization.
☐ 1 ☐ 2 ☐ 3 ☐ 4 ☐ 5

SI5 - I use Artificial Intelligence due to influence of social media.
☐ 1 ☐ 2 ☐ 3 ☐ 4 ☐ 5

SI6 - I use Artificial Intelligence due to my surroundings have use it a lot..
☐ 1 ☐ 2 ☐ 3 ☐ 4 ☐ 5

SI7 - I use Artificial Intelligence to improve my self-image.
☐ 1 ☐ 2 ☐ 3 ☐ 4 ☐ 5

FC1 - I have the necessary facilities to access Artificial Intelligence.
☐ 1 ☐ 2 ☐ 3 ☐ 4 ☐ 5

FC2 - I have enough ability and knowledge to use Artificial Intelligence..
☐ 1 ☐ 2 ☐ 3 ☐ 4 ☐ 5

FC3 - Some Experts are available to help me when I have trouble using Artificial Intelligence.
☐ 1 ☐ 2 ☐ 3 ☐ 4 ☐ 5

FC4 - Artificial Intelligence can help work effectively.
☐ 1 ☐ 2 ☐ 3 ☐ 4 ☐ 5

IT1 - I am concerned about internet security when using Artificial Intelligence.
☐ 1 ☐ 2 ☐ 3 ☐ 4 ☐ 5

IT2 - I am concerned about online transaction reliability with Artificial Intelligence.
☐ 1 ☐ 2 ☐ 3 ☐ 4 ☐ 5

IT3 - I am concerned about trustworthiness when using Artificial Intelligence.
☐ 1 ☐ 2 ☐ 3 ☐ 4 ☐ 5

IT4 - I am concerned about online privacy when using Artificial Intelligence.
☐ 1 ☐ 2 ☐ 3 ☐ 4 ☐ 5

IT5 - I am concerned the effectiveness of internet when using Artificial Intelligence.
☐ 1 ☐ 2 ☐ 3 ☐ 4 ☐ 5

SQ1 - Artificial Intelligence can reduce the errors.
☐ 1 ☐ 2 ☐ 3 ☐ 4 ☐ 5

SQ2 - Artificial Intelligence provides fast response time.
☐ 1 ☐ 2 ☐ 3 ☐ 4 ☐ 5

SQ3 - Artificial Intelligence is reliable.
☐ 1 ☐ 2 ☐ 3 ☐ 4 ☐ 5

SQ4 - Artificial Intelligence can be used at any time.
☐ 1 ☐ 2 ☐ 3 ☐ 4 ☐ 5

SQ5 - Artificial Intelligence functions well to my work.
☐ 1 ☐ 2 ☐ 3 ☐ 4 ☐ 5

TSF1 - Artificial Intelligence can save my time.
☐ 1 ☐ 2 ☐ 3 ☐ 4 ☐ 5

TSF2 - Artificial Intelligence can reduce my routine tasks.
☐ 1 ☐ 2 ☐ 3 ☐ 4 ☐ 5

TSF3 - Artificial Intelligence can provide me the information.
☐ 1 ☐ 2 ☐ 3 ☐ 4 ☐ 5

TSF4 - Artificial Intelligence can reduce the manual human work.
☐ 1 ☐ 2 ☐ 3 ☐ 4 ☐ 5

TSE1 - I am confident in my ability to use Artificial Intelligence at work.
☐ 1 ☐ 2 ☐ 3 ☐ 4 ☐ 5

TSE2 - I am confident in my ability to use Artificial Intelligence automating to replace my routine accounting task.
☐ 1 ☐ 2 ☐ 3 ☐ 4 ☐ 5

TSE3 - I am confident in my ability to use Artificial Intelligence to generate financial reports.
☐ 1 ☐ 2 ☐ 3 ☐ 4 ☐ 5

TSE4 - I am confident in my ability to use Artificial Intelligence into company existing accounting software.
☐ 1 ☐ 2 ☐ 3 ☐ 4 ☐ 5

TSE5 - I am confident in my ability to use Artificial Intelligence on my smartphone or tablet.
☐ 1 ☐ 2 ☐ 3 ☐ 4 ☐ 5

EA1 - I am aware of Artificial Intelligence technology.
☐ 1 ☐ 2 ☐ 3 ☐ 4 ☐ 5

EA2 - I am aware of the concept of Artificial Intelligence.
☐ 1 ☐ 2 ☐ 3 ☐ 4 ☐ 5

EA3 - I am aware of the purpose of using Artificial Intelligence in the workplace.
☐ 1 ☐ 2 ☐ 3 ☐ 4 ☐ 5

EA4 - I agree with the benefits brought by Artificial Intelligence.
☐ 1 ☐ 2 ☐ 3 ☐ 4 ☐ 5

EA5 - I feel I know all the usage of Artificial Intelligence.
☐ 1 ☐ 2 ☐ 3 ☐ 4 ☐ 5

PI1 - I will try a new technology when I hear of it.
☐ 1 ☐ 2 ☐ 3 ☐ 4 ☐ 5

PI2 - I will be the first user to try new technologies among my peers.
☐ 1 ☐ 2 ☐ 3 ☐ 4 ☐ 5

PI3 - I am not hesitant to experiment with new technology.
☐ 1 ☐ 2 ☐ 3 ☐ 4 ☐ 5

PI4 - I enjoy exploring new technology.
☐ 1 ☐ 2 ☐ 3 ☐ 4 ☐ 5

IU1 - I intend to continue using Artificial Intelligence software in the future.
☐ 1 ☐ 2 ☐ 3 ☐ 4 ☐ 5

IU2 - I will always try to use Artificial Intelligence in my daily professional work.
☐ 1 ☐ 2 ☐ 3 ☐ 4 ☐ 5

IU3 - I plan to use Artificial Intelligence frequently.
☐ 1 ☐ 2 ☐ 3 ☐ 4 ☐ 5

Appendix V

*WebPower output*


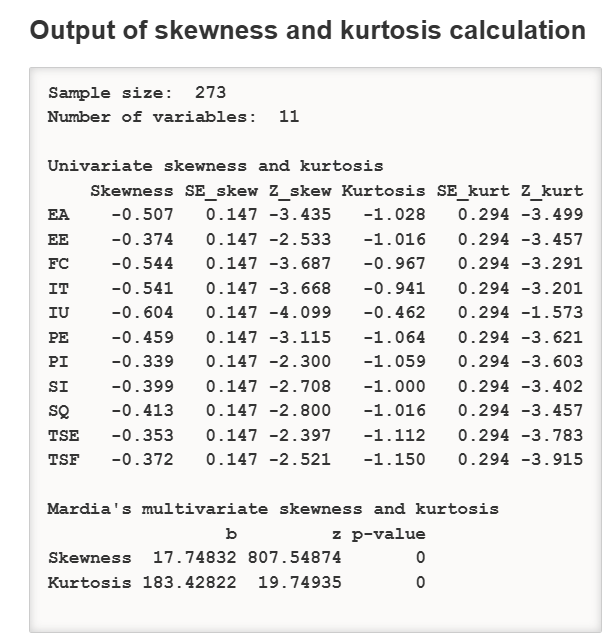


Appendix VI

*Univariate Skewness and Kurtosis calculation results (from SPSS)*

| *Descriptive Statistics* |
| --- |

| Constructs | N  Statistic | Minimum  Statistic | Maximum  Statistic | Mean  Statistic | Std. Deviation  Statistic | Skewness | | Kurtosis | | |
| --- | --- | --- | --- | --- | --- | --- | --- | --- | --- | --- |
|  |  |  |  |  |  | Statistic | Std. Error | Statistic | Std. Error |  |
| PE1 | 273 | 1 | 5 | 3.51 | 1.386 | -.550 | .147 | -1.012 | .294 |  |
| PE2 | 273 | 1 | 5 | 3.37 | 1.239 | -.347 | .147 | -.816 | .294 |  |
| PE3 | 273 | 1 | 5 | 3.40 | 1.319 | -.418 | .147 | -.960 | .294 |  |
| PE4 | 273 | 1 | 5 | 3.41 | 1.185 | -.313 | .147 | -.800 | .294 |  |
| PE5 | 273 | 1 | 5 | 3.51 | 1.167 | -.500 | .147 | -.569 | .294 |  |
| EE1 | 273 | 1 | 5 | 3.32 | 1.314 | -.329 | .147 | -1.014 | .294 |  |
| EE2 | 273 | 1 | 5 | 3.21 | 1.213 | -.213 | .147 | -.872 | .294 |  |
| EE3 | 273 | 1 | 5 | 3.25 | 1.300 | -.347 | .147 | -.927 | .294 |  |
| EE4 | 273 | 1 | 5 | 3.27 | 1.215 | -.191 | .147 | -.967 | .294 |  |
| SI1 | 273 | 1 | 5 | 3.30 | 1.374 | -.331 | .147 | -1.150 | .294 |  |
| SI2 | 273 | 1 | 5 | 3.14 | 1.268 | -.173 | .147 | -.941 | .294 |  |
| SI3 | 273 | 1 | 5 | 3.37 | 1.193 | -.281 | .147 | -.683 | .294 |  |
| SI4 | 273 | 1 | 5 | 3.35 | 1.179 | -.210 | .147 | -.783 | .294 |  |
| SI5 | 273 | 1 | 5 | 3.29 | 1.210 | -.321 | .147 | -.696 | .294 |  |
| SI6 | 273 | 1 | 5 | 3.40 | 1.190 | -.290 | .147 | -.754 | .294 |  |
| SI7 | 273 | 1 | 5 | 3.20 | 1.218 | -.151 | .147 | -.850 | .294 |  |
| FC1 | 273 | 1 | 5 | 3.37 | 1.308 | -.383 | .147 | -.953 | .294 |  |
| FC2 | 273 | 1 | 5 | 3.31 | 1.246 | -.302 | .147 | -.912 | .294 |  |
| FC3 | 273 | 1 | 5 | 3.33 | 1.255 | -.273 | .147 | -.931 | .294 |  |
| FC4 | 273 | 1 | 5 | 3.37 | 1.285 | -.417 | .147 | -.899 | .294 |  |
| IT1 | 273 | 1 | 5 | 3.46 | 1.364 | -.511 | .147 | -.981 | .294 |  |
| IT2 | 273 | 1 | 5 | 3.34 | 1.199 | -.429 | .147 | -.630 | .294 |  |
| IT3 | 273 | 1 | 5 | 3.27 | 1.176 | -.330 | .147 | -.583 | .294 |  |
| IT4 | 273 | 1 | 5 | 3.34 | 1.196 | -.285 | .147 | -.753 | .294 |  |
| IT5 | 273 | 1 | 5 | 3.29 | 1.246 | -.391 | .147 | -.750 | .294 |  |
| SQ1 | 273 | 1 | 5 | 3.40 | 1.355 | -.452 | .147 | -1.018 | .294 |  |
| SQ2 | 273 | 1 | 5 | 3.37 | 1.213 | -.325 | .147 | -.871 | .294 |  |
| SQ3 | 273 | 1 | 5 | 3.25 | 1.235 | -.225 | .147 | -.892 | .294 |  |
| SQ4 | 273 | 1 | 5 | 3.31 | 1.252 | -.339 | .147 | -.889 | .294 |  |
| SQ5 | 273 | 1 | 5 | 3.33 | 1.255 | -.381 | .147 | -.876 | .294 |  |
| TSF1 | 273 | 1 | 5 | 3.32 | 1.377 | -.408 | .147 | -1.110 | .294 |  |
| TSF2 | 273 | 1 | 5 | 3.35 | 1.207 | -.265 | .147 | -.819 | .294 |  |
| TSF3 | 273 | 1 | 5 | 3.45 | 1.185 | -.391 | .147 | -.703 | .294 |  |
| TSF4 | 273 | 1 | 5 | 3.44 | 1.233 | -.400 | .147 | -.793 | .294 |  |
| TSE1 | 273 | 1 | 5 | 3.44 | 1.316 | -.414 | .147 | -.996 | .294 |  |
| TSE2 | 273 | 1 | 5 | 3.29 | 1.118 | -.273 | .147 | -.560 | .294 |  |
| TSE3 | 273 | 1 | 5 | 3.30 | 1.130 | -.304 | .147 | -.517 | .294 |  |
| TSE4 | 273 | 1 | 5 | 3.32 | 1.139 | -.176 | .147 | -.722 | .294 |  |
| TSE5 | 273 | 1 | 5 | 3.26 | 1.183 | -.315 | .147 | -.741 | .294 |  |
| EA1 | 273 | 1 | 5 | 3.48 | 1.337 | -.538 | .147 | -.914 | .294 |  |
| EA2 | 273 | 1 | 5 | 3.34 | 1.197 | -.392 | .147 | -.668 | .294 |  |
| EA3 | 273 | 1 | 5 | 3.39 | 1.208 | -.391 | .147 | -.763 | .294 |  |
| EA4 | 273 | 1 | 5 | 3.39 | 1.177 | -.343 | .147 | -.673 | .294 |  |
| EA5 | 273 | 1 | 5 | 3.25 | 1.202 | -.158 | .147 | -.787 | .294 |  |
| PI1 | 273 | 1 | 5 | 3.31 | 1.276 | -.419 | .147 | -.839 | .294 |  |
| PI2 | 273 | 1 | 5 | 3.20 | 1.169 | -.148 | .147 | -.654 | .294 |  |
| PI3 | 273 | 1 | 5 | 3.31 | 1.185 | -.162 | .147 | -.859 | .294 |  |
| PI4 | 273 | 1 | 5 | 3.36 | 1.210 | -.334 | .147 | -.690 | .294 |  |
| IU1 | 273 | 1 | 5 | 3.59 | 1.342 | -.717 | .147 | -.619 | .294 |  |
| IU2 | 273 | 1 | 5 | 3.32 | 1.090 | -.317 | .147 | -.531 | .294 |  |
| IU3 | 273 | 1 | 5 | 3.27 | 1.148 | -.304 | .147 | -.584 | .294 |  |
| Valid N (listwise) | 273 |  |  |  |  |  |  |  |  |  |

**References**

[1] R. e. Almashawreh, M. Talukder, S. K. Charath, and M. I. Khan, "AI adoption in Jordanian SMEs: The influence of technological and organizational orientations," *Global Business Review,* p. 09721509241250273, 2024.

[2] S. Badghish and Y. A. Soomro, "Artificial intelligence adoption by SMEs to achieve sustainable business performance: application of technology–organization–environment framework," *Sustainability,* vol. 16, no. 5, p. 1864, 2024.

[3] A. M. Nascimento and F. d. S. Meirelles, "Factors influencing the adoption intention of artificial intelligence in small businesses," 2022.

[4] J. Schwaeke, A. Peters, D. K. Kanbach, S. Kraus, and P. Jones, "The new normal: The status quo of AI adoption in SMEs," *Journal of small business management,* vol. 63, no. 3, pp. 1297-1331, 2025.

[5] S. Lada *et al.*, "Determining factors related to artificial intelligence (AI) adoption among Malaysia's small and medium-sized businesses," *Journal of Open Innovation: Technology, Market, and Complexity,* vol. 9, no. 4, p. 100144, 2023.

[6] A. S. Jameel, S. A. Harjan, and A. R. Ahmad, "Behavioral intentions to use artificial intelligence among managers in small and medium enterprises," in *International Conference on Advances in Communication Technology and Computer Engineering*, 2023, vol. 2814, no. 1: AIP Publishing LLC, p. 020006.

[7] S. S. Ingalagi, R. R. Mutkekar, and P. Kulkarni, "Artificial Intelligence (AI) adaptation: Analysis of determinants among Small to Medium-sized Enterprises (SME’s)," in *IOP Conference Series: Materials Science and Engineering*, 2021, vol. 1049, no. 1: IOP Publishing, p. 012017.

[8] D. Mam, N. Por, and B. Kep, "Technology Adoption Among Women in Cambodia's SMEs: Influencing Factors, Benefits, and Coping Strategies," *Journal of Mathematics Instruction, Social Research and Opinion,* vol. 4, no. 1, pp. 175-194, 2025.

[9] M. A. Kwarteng, A. Ntsiful, L. F. P. Diego, and P. Novák, "Extending UTAUT with competitive pressure for SMEs digitalization adoption in two European nations: a multi-group analysis," *Aslib Journal of Information Management,* vol. 76, no. 5, pp. 842-868, 2024.

[10] T. Wijaya and S. Budiman, "The intention of adopting information technology for SMES in Special Region of Yogyakarta," *JDM (Jurnal Dinamika Manajemen),* vol. 10, no. 2, pp. 205-215, 2019.

[11] M. I. Effendi, D. Sugandini, and Y. Istanto, "Social media adoption in SMEs impacted by COVID-19: The TOE model," *The Journal of Asian Finance, Economics and Business,* vol. 7, no. 11, pp. 915-925, 2020.

[12] E. Triandini, I. Wijaya, and I. K. P. Suniantara, "Analysis of technology adoption by SMEs using technology organization environment model," *Journal of System and Management Sciences,* vol. 13, no. 2, pp. 225-240, 2023.
